# Topographic, soil, and climate drivers of drought sensitivity in forests and shrublands of the Pacific Northwest, USA

**DOI:** 10.1038/s41598-020-75273-5

**Published:** 2020-10-28

**Authors:** Jennifer M. Cartwright, Caitlin E. Littlefield, Julia L. Michalak, Joshua J. Lawler, Solomon Z. Dobrowski

**Affiliations:** 1 Lower Mississippi-Gulf Water Science Center, U.S. Geological Survey, Nashville, USA; 2 Rubenstein School of Environment and Natural Resources, University of Vermont, Burlington, USA; 3 School of Environmental and Forest Sciences, University of Washington, Seattle, USA; 4 Department of Forest Management, University of Montana, Missoula, MT USA

**Keywords:** Climate-change ecology, Ecophysiology, Climate-change impacts, Natural variation in plants

## Abstract

Climate change is anticipated to increase the frequency and intensity of droughts, with major impacts to ecosystems globally. Broad-scale assessments of vegetation responses to drought are needed to anticipate, manage, and potentially mitigate climate-change effects on ecosystems. We quantified the drought sensitivity of vegetation in the Pacific Northwest, USA, as the percent reduction in vegetation greenness under droughts relative to baseline moisture conditions. At a regional scale, shrub-steppe ecosystems—with drier climates and lower biomass—showed greater drought sensitivity than conifer forests. However, variability in drought sensitivity was considerable within biomes and within ecosystems and was mediated by landscape topography, climate, and soil characteristics. Drought sensitivity was generally greater in areas with higher elevation, drier climate, and greater soil bulk density. Ecosystems with high drought sensitivity included dry forests along ecotones to shrublands, Rocky Mountain subalpine forests, and cold upland sagebrush communities. In forests, valley bottoms and areas with low soil bulk density and high soil available water capacity showed reduced drought sensitivity, suggesting their potential as drought refugia. These regional-scale drought-sensitivity patterns discerned from remote sensing can complement plot-scale studies of plant physiological responses to drought to help inform climate-adaptation planning as drought conditions intensify.

## Introduction

Climate change is projected to increase drought frequency and intensity in many parts of the world^1–3^. Drought intensification combined with hotter temperatures may lead to forest decline through mortality^4,5^ and recruitment failure^6^, impacting primary production and ecosystem services such as wildlife habitat and carbon storage^7,8^. To predict and manage these changes at regional scales, assessments of ecosystem responses to drought are needed across broad climate-vegetation types (biomes) to complement plot-scale studies on plant physiological responses to drought.

Vegetation responses to drought are controlled by physical processes at the landscape level such as soil water routing and retention, groundwater interactions, and evaporative demand^9–12^ and by plant community composition and stress-tolerance thresholds of species, populations, and individuals^13–15^. Variability across spatial and temporal scales in plant responses to drought can include differences in water-use efficiency, stomatal regulation, hydraulic characteristics, and structural adjustments to root networks and leaf area^7,15–18^. For example, plant species and functional groups differ in their approaches to balancing trade-offs between regulating leaf-water potentials and maintaining stomatal conductance, with consequences for susceptibility to drought-induced carbon starvation and/or hydraulic failure^15,19^.

Variability in plant responses to drought can influence aboveground productivity, which in turn produces spectral differences discernible in satellite imagery. Remote sensing has enabled regional- and global-scale assessments of vegetation responses to drought that generally agree with information from other sources such as land-surface models, dendrochronology, flux-tower data, and field observations^18,20–22^. For example, annually integrated Enhanced Vegetation Index (EVI) from the Moderate-resolution Imaging Spectroradiometer (MODIS) is strongly correlated with field measurements of aboveground net primary productivity (ANPP), allowing its use as a proxy for ANPP^18,23,24^. For representing ANPP across a range of biomes, EVI is preferable to other spectral indices such as Normalized Difference Vegetation Index (NDVI), due to the tendency of NDVI to saturate in high-biomass areas^25,26^. Use of EVI to assess how vegetation productivity responds to droughts helps overcome the limited spatial availability and inconsistencies (both among and within sites) associated with field-based productivity measurements^23^. However, regional-scale studies based on remote sensing can only detect vegetation responses to drought that produce canopy-level spectral changes, a subset of the potentially important responses that may also include physiological shifts and below-ground changes^27^.

Although remote sensing provides powerful tools to identify broad-scale vegetation drought responses, anticipating the ecosystem effects of drought intensification requires improved understanding of the underlying biophysical processes that shape drought impacts within and across biomes. Some factors governing drought responses may be greatly affected by climate change (e.g. regional temperature and moisture gradients), whereas others will be more stable through time (e.g. topographic and soil characteristics). Previous studies have characterized large-scale spatial patterns of vegetation responses to meteorological variability and have linked these responses to patterns of biomass and climate, generally finding stronger drought impacts in areas where productivity is water-limited^18,20,26,28–30^, but have not accounted for within-biome variability based on the effects of topography, soil, and landscape hydrology. These landscape characteristics produce spatial heterogeneity of soil water availability and evaporative demand during droughts, owing to processes such as soil drainage and water retention, water storage in weathered bedrock, lateral routing of soil water and groundwater, cold-air pooling, and shading from solar radiation^11,12,31,32^. Such landscape characteristics can thus affect vegetation community structure and plant physiological responses to drought^5,6^ including vulnerability to drought-induced mortality^12,33^, and may produce localized areas of hydrologic buffering from climate variability and drought impacts, i.e., hydrologic refugia^11,31^. Understanding the biophysical processes that shape ecosystem responses to drought can help land managers better anticipate—and potentially mitigate—biodiversity and ecosystem-service losses as droughts intensify.

We examined the landscape and climate controls on vegetation responses to drought across two prevalent biomes in the Pacific Northwest of the USA: conifer forest and shrub steppe. This region includes diverse topography, climates, and vegetation (from temperate rainforest to semi-arid shrublands), allowing us to compare drought-response patterns across large gradients of energy and water availability. Comparison across large climate gradients is useful because relationships between landscape topography and vegetation responses to drought can vary depending on climate conditions^11^. Like many areas of western North America, the Pacific Northwest region is projected to experience continued snowpack reductions, hotter and longer summers, and the intensification of seasonal droughts^2,34,35^.

We conceptualized drought sensitivity as the reduction in ecosystem productivity in response to drought and quantified that sensitivity using EVI, which is representative of ANPP^18,23,24^. This conceptualization of drought sensitivity is slightly different from—and complementary to—how other studies have assessed drought sensitivity, i.e., as the slope or correlation coefficient of the relationship between a meteorological metric and a vegetation metric such as EVI, NDVI, or ANPP^18,20,28,30^. Correlation- or regression-based drought-sensitivity metrics typically represent vegetation responses across the full range of variability in climatic moisture conditions at a given site, from wet years to dry years^20,30^. By considering differences in vegetation metrics under wet meteorological conditions relative to long-term average conditions, such correlation- or regression-based metrics are not strictly focused on drought per se, but rather on overall climatic variability. Our complementary approach focused only on drought effects, by comparing vegetation conditions under droughts of varying intensity levels versus under long-term average meteorological conditions. This approach enabled the differentiation of vegetation responses to severe versus moderate droughts and was not directly affected by vegetation responses to pluvial periods.

We used machine-learning models to investigate the spatial patterns of drought sensitivity related to factors that affect water availability, including climate, landscape topography, soil characteristics, and hydrologic indicators. Because previous studies have found stronger vegetation responses to climate variability with decreasing climatic wetness and biomass^20,28^, we anticipated that drought sensitivity would be greater in the shrub-steppe biome than in conifer forest. We also hypothesized that drought sensitivity would be reduced by soil characteristics that increase water infiltration and storage and by topographic features that concentrate runoff or suppress evaporative demand^11,31,33^. By examining the spatial drivers underlying drought sensitivity across biomes and drought-intensity levels, this study improves our ability to identify drought-sensitive ecosystems and anticipate ecosystem response to drought intensification under climate change.

## Results

### Regional drought sensitivity patterns

**Figure 1:**
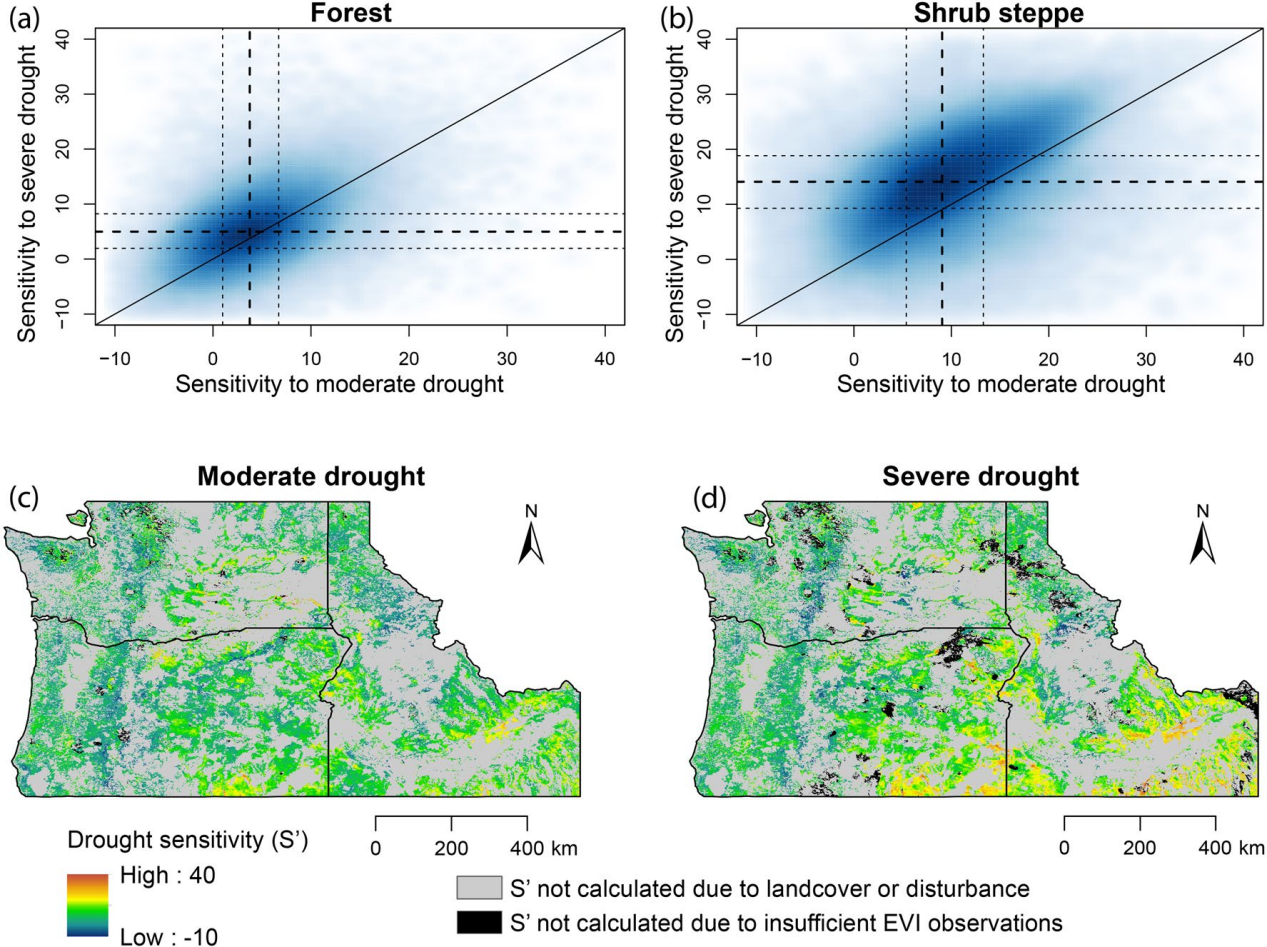
Drought sensitivity (*S’*) for Pacific Northwest forests **(a)** and shrub-steppe areas **(b)**, depicted as density plots of *S’* distributions in response to moderate drought and severe drought. Solid diagonal lines in **(a)** and **(b)** represent 1:1 correspondence of moderate-drought sensitivity and severe-drought sensitivity. Thick dashed lines indicate median drought-sensitivity values; thinner dashed lines represent 25th and 75th percentile values. Maps in **(c)** and **(d)** depict sensitivity to moderate drought and severe drought, respectively, for all forest and shrub-steppe pixels analyzed. Landcover types, state names, and geographic features are available in fig. S1. Drought sensitivity was calculated using Enhanced Vegetation Index (EVI) data obtained from the Moderate-resolution Imaging Spectroradiometer (MODIS) from EarthData Search^59^. Data processing was performed in the R statistical environment^66^. Maps were created using Esri ArcGIS Desktop v.10.4.1^74^.

Drought sensitivity (*S’*) was generally greater in shrub-steppe areas than in forest (Fig. 1a,b), evident in geographic patterns of greater *S’* values in sagebrush-dominated areas of southeastern Oregon and southern Idaho relative to forested parts of the study area such as western Washington and Oregon (Fig. 1c,d; see supplementary Fig. S1 for a map of landcover types, state names, and geographic features). In forests, the median reduction of EVI under drought relative to baseline conditions (i.e., drought sensitivity) was 3.75 and 4.98% (for moderate and severe drought, respectively), compared to 9.05 and 14.11% for shrub-steppe areas. Sensitivities to moderate and severe drought were positively correlated (Spearman’s ρ = 0.50 and 0.55 for forest and steppe, respectively, both p < 0.001). Shrub-steppe areas showed greater differential sensitivity to severe compared to moderate drought than did forests: in shrub-steppe areas, sensitivity to severe drought (*S’*_*sev*_) exceeded sensitivity to moderate drought (*S’*_*mod*_) in roughly four out of five pixels, by 6.8 units (percentage points) on average, whereas in forested areas, *S’*_*sev*_ exceeded *S’*_*mod*_ in fewer than two-thirds of pixels, by 4.2 units on average.

### Climate and landscape influences on drought sensitivity

**Figure 2:**
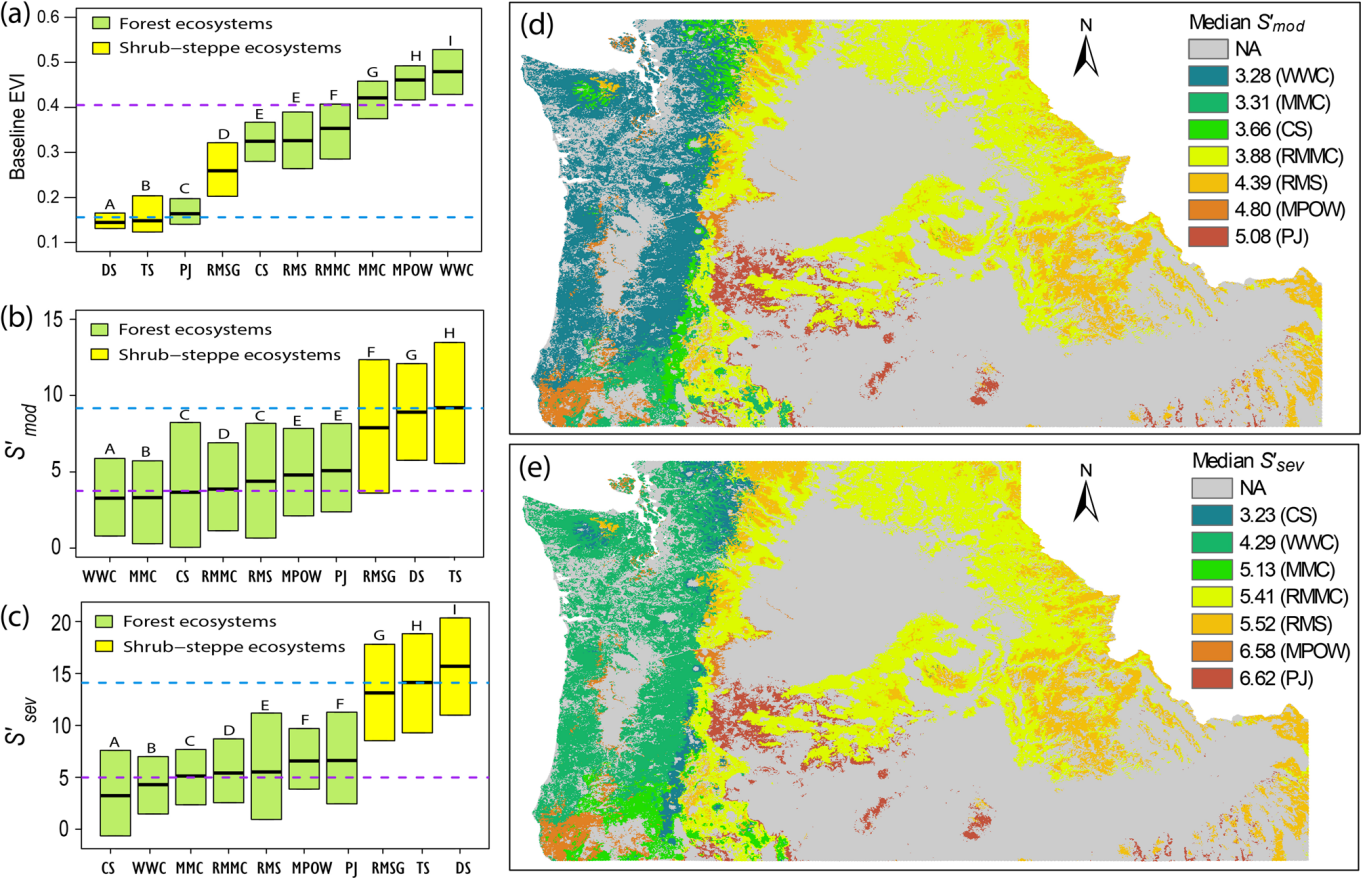
**(a)** Baseline Enhanced Vegetation Index (EVI) and sensitivity to **(b)** moderate drought (S’_*mod*_) and **(c)** severe drought (S’_*sev*_) by ecosystem types. Boxes show medians and interquartile ranges; dashed lines represent medians across all forest types (purple) and shrub-steppe types (blue); different letters represent significant differences from a pairwise t-test. Maps show median sensitivity to **(d)** moderate drought and **(e)** severe drought by forest ecosystem type, with median values assigned to all pixels mapped as that forest type; note this includes pixels not used for analysis due to disturbances. *CS* cascade subalpine forest, *DS* dwarf sagebrush, *MMC* Mediterranean mixed conifer forest, *MPOW* Mediterranean pine-oak woodland, *PJ* pinyon-juniper woodland, *RMMC* Rocky Mountain mixed conifer forest, *RMS* Rocky Mountain subalpine forest, *RMSG* Rocky Mountain shrubland and grassland, *TS* tall sagebrush, *WWC* western wet conifer forest (see supplementary Table S1). Drought sensitivity was calculated using EVI data obtained from the Moderate-resolution Imaging Spectroradiometer (MODIS) from EarthData Search^59^. Data processing was performed in the R statistical environment^66^. Maps were created using Esri ArcGIS Desktop v.10.4.1^74^.

Within both forest and shrub-steppe biomes, ecosystem types differed in both their baseline EVI and their drought sensitivity (Fig. 2). Shrub-steppe ecosystems tended to have lower baseline EVI than forest ecosystems, with the exception of pinyon-juniper (*Pinus-Juniperus*) woodlands (Fig. 2a). Geographic patterns of baseline EVI largely reflected gradients of actual evapotranspiration (AET) and climatic water deficit (Spearman’s ρ = 0.85 and -0.81, respectively, both p < 0.001), indicating that—as expected—baseline EVI is strongly associated with ecosystem productivity and is constrained by climatic water limitation. Secondarily, baseline EVI decreased somewhat with elevation (Spearman’s ρ = -0.41, p < 0.001), reflecting the lower productivity of subalpine forest ecosystems relative to lower-elevation coastal forests west of the Cascade Mountains (Fig. 2; see supplementary Fig. S1 for geographic features of the study area).

**Figure 3:**
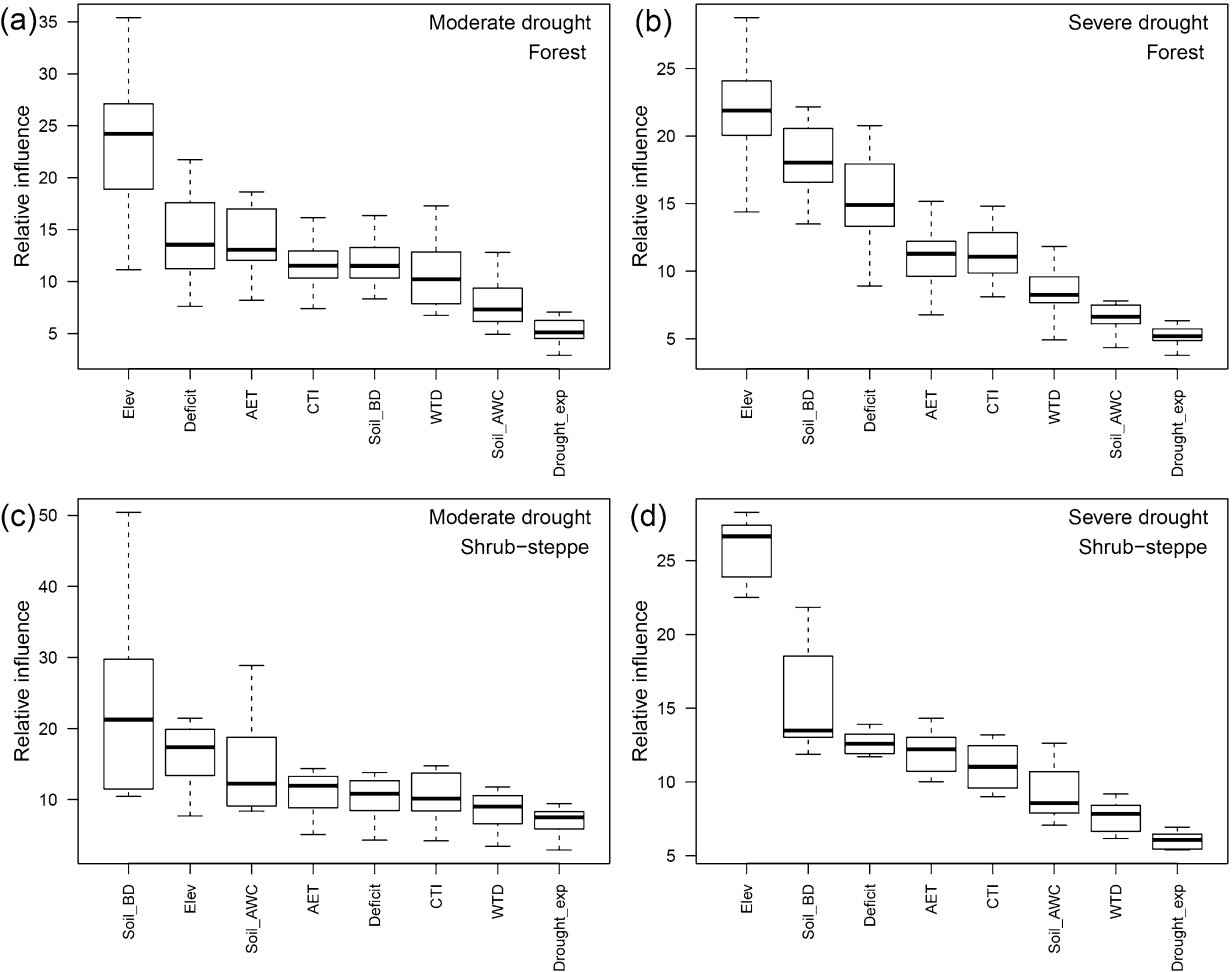
Distribution of relative influence values for predictor variables (defined in Table 1) in boosted regression tree (BRT) models for drought sensitivity in **(a)** forests under moderate drought, **(b)** forests under severe drought, **(c)** shrub-steppe under moderate drought, and **(d)** shrub-steppe under severe drought. Boxplots represent variability across 20 models runs, showing interquartile ranges and medians (boxes) and minimum and maximum values (whiskers). This figure was created in the R statistical environment^66^.

Variability in drought sensitivity within ecosystem types was substantial (Fig. 2b,c) and ecosystem type contributed little to preliminary models (supplementary Fig. S2), highlighting the importance of climate and landscape (i.e., topographic and soil) variables in shaping drought sensitivity. Large within-ecosystem variability (Fig. 2b,c) and relative influence patterns (Fig. 3, supplementary Fig. S2) suggest that differences in drought sensitivity among ecosystems were driven primarily by ecosystem-level differences in climate and landscape characteristics.

**Figure 4:**
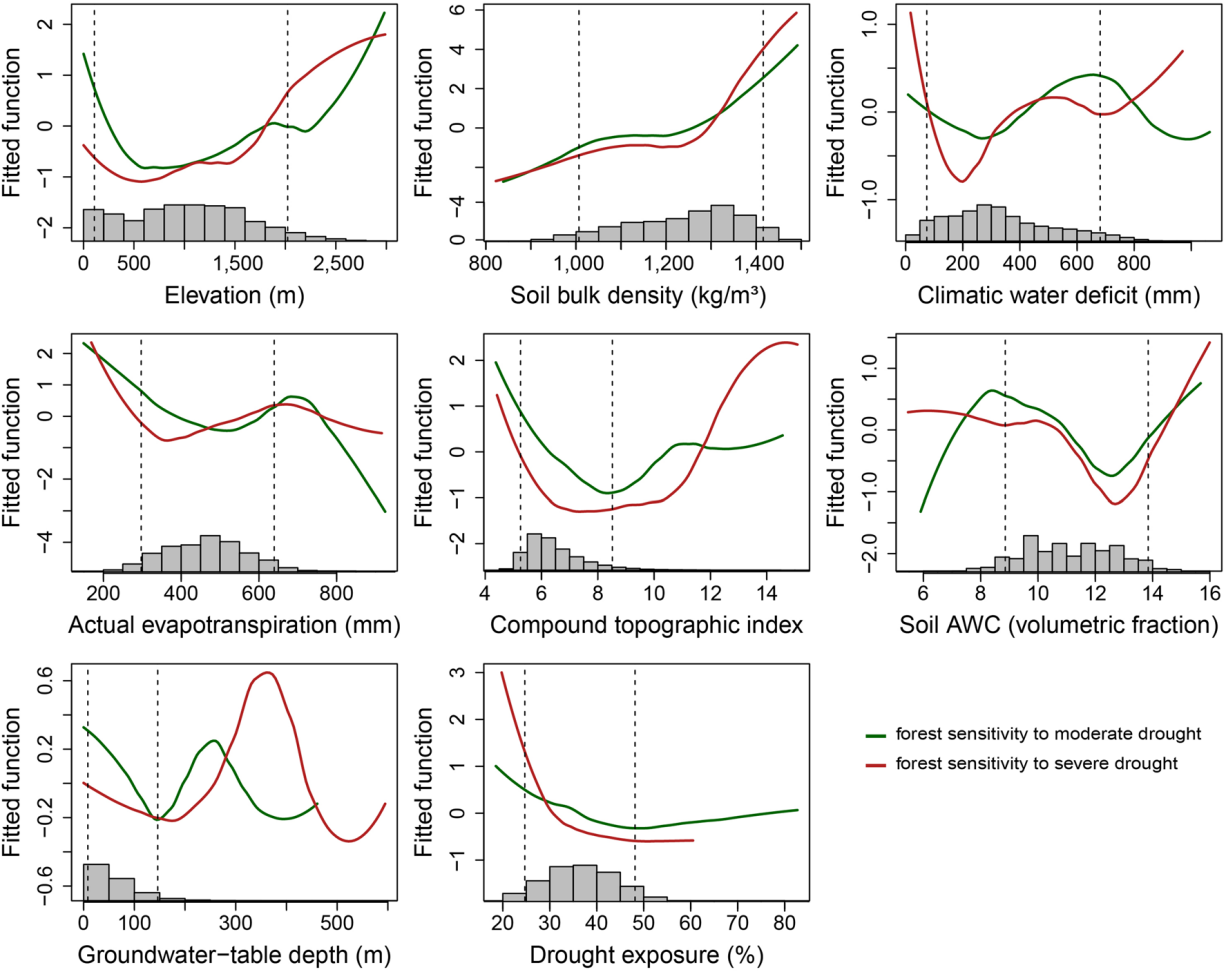
Partial-dependence plots showing marginal influence on forest drought sensitivity (*S’*) of boosted-regression tree model predictors (defined in Table 1). Lines represent smoothed averages across 20 model runs; see supplementary Figs S3 through S6. Histograms show distributions of predictors across all forest pixels used in modeling. Because of stochastic instability at the extreme ends of predictor variables (supplementary modeling results), our interpretation of partial-dependence plots focuses on the regions between the 5th and 95th percentiles of each predictor (vertical dashed lines). This figure was created in the R statistical environment^66^.

**Figure 5:**
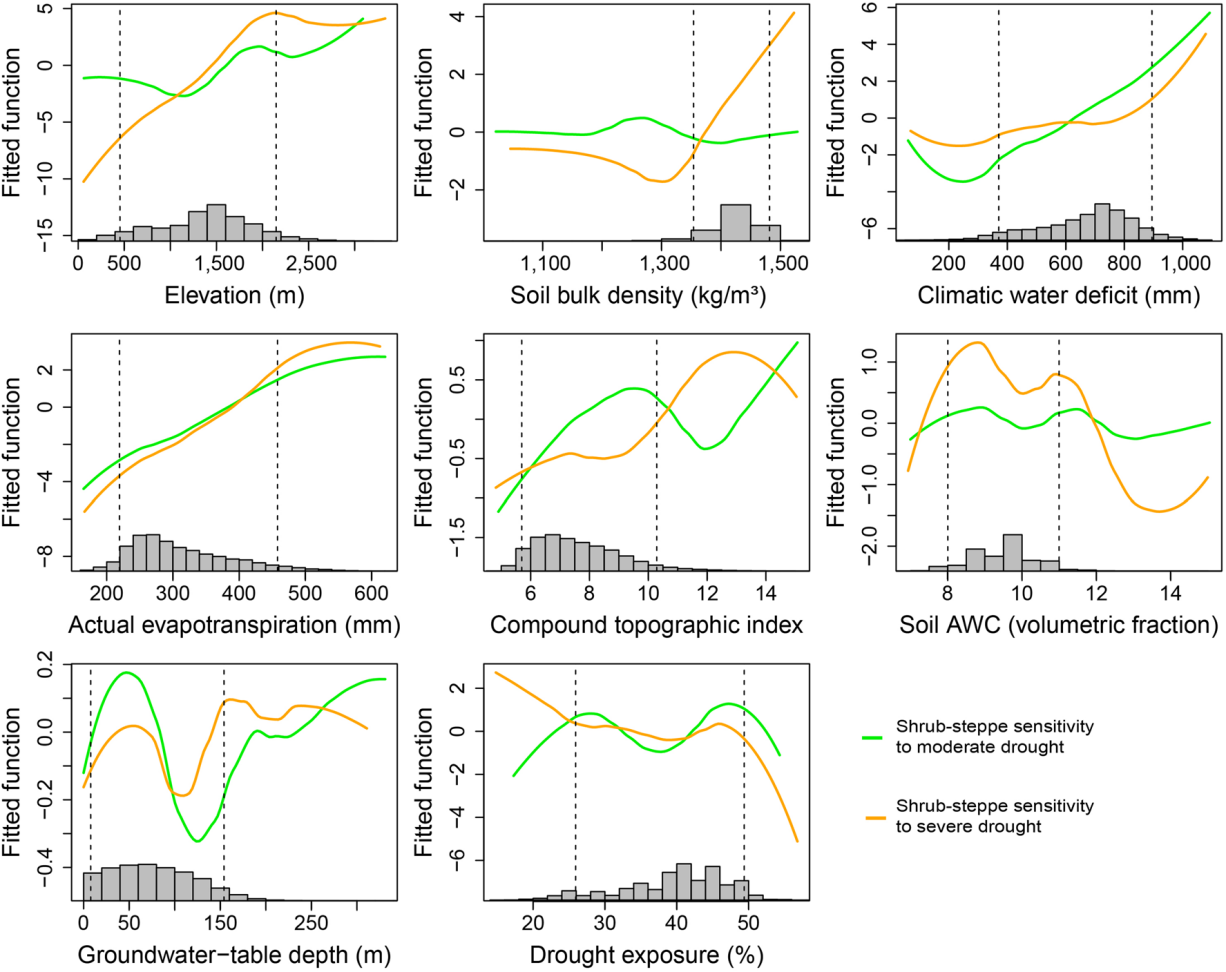
Partial-dependence plots showing marginal influence on shrub-steppe drought sensitivity (*S’*) of boosted-regression tree model predictors (defined in Table 1). Lines represent smoothed averages across 20 model runs; see supplementary Figs S3 through S6. Histograms show distributions of predictors across all shrub-steppe pixels used in modeling. Because of stochastic instability at the extreme ends of predictor variables (supplementary modeling results), our interpretation of partial-dependence plots focuses on the regions between the 5th and 95th percentiles of each predictor (vertical dashed lines). This figure was created in the R statistical environment^66^.

Elevation was a dominant driver of *S’* patterns, with climate variables and soil bulk density also playing relatively strong roles in explaining spatial patterns in *S’* (Fig. 3). In forests located higher than 500 m above sea level, *S’* generally increased with elevation (Fig. 4, supplementary Figs. S3 and S4), reflected in relatively high *S’* for Rocky Mountain subalpine forests (Fig. 2). Shrub-steppe sensitivity to severe drought also increased strongly with elevation (Fig. 5, supplementary Figs. S5 and S6). Among shrub-steppe ecosystems, drought sensitivity was greater in sagebrush types (i.e., dwarf and tall sagebrush) than in Rocky Mountain shrubland and grassland (Fig. 2). These sagebrush ecosystems occur at higher elevations than Rocky Mountain shrubland and grassland, have more constrained productivity (lower AET) and greater water limitation (climatic water deficit), and are more prevalent in convergent environments (i.e., with high compound topographic index; CTI).

Drought sensitivity generally increased with increasing water limitation in shrub-steppe areas and in forests with climatic water deficit > 300 mm (Figs. 4 and 5). Maps of median values of drought sensitivity by forest type show an overall geographic pattern of greater sensitivity in the drier forest ecosystem types east of the Cascade Mountains, i.e., pinyon-juniper woodland, Rocky Mountain subalpine, and Rocky Mountain mixed-conifer forests (Fig. 2). Sensitivity was greatest for pinyon-juniper woodland—the driest forest type in the region—and was also relatively high for the Mediterranean pine-oak (*Pinus-Quercus*) woodlands, whereas wet coastal forests showed generally low drought sensitivity.

Soil bulk density showed high relative influence in 3 of 4 models (i.e., in forests under severe drought and in shrub-steppe ecosystems under both moderate and severe droughts, Fig. 3), with *S’* generally higher in areas with more compacted soils (Figs. 4 and 5). Although soil available water capacity (AWC) generally had lower relative influence, *S’* was greatest in soils with lowest soil AWC under both drought-intensity levels in forests and in shrub-steppe areas under severe droughts. Areas of greater topographic convergence (higher CTI) such as valley bottoms and riparian areas showed reduced *S’* in forest (Fig. 4) but not in shrub-steppe (Fig. 5). Convergent areas are generally associated with deep soils and shallow groundwater availability^36^. Contrary to expectations, however, none of the models showed compelling evidence for reduced drought sensitivity in areas of shallow groundwater, which may be attributable to underrepresentation of localized shallow groundwater availability at sub-kilometer scales (part 2 in supplementary material).

Several other variables with plausible connections to drought sensitivity contributed little toward explaining the observed drought-sensitivity patterns. The relative influence of drought exposure on *S’* patterns was universally low for all models (Fig. 3), however, forest *S’* tended to be greater in areas that had experienced the lowest previous drought exposure. Topographic shading metrics had low relative influence in preliminary models (supplementary Fig. S2) so were not used in final models.

**Figure 6:**
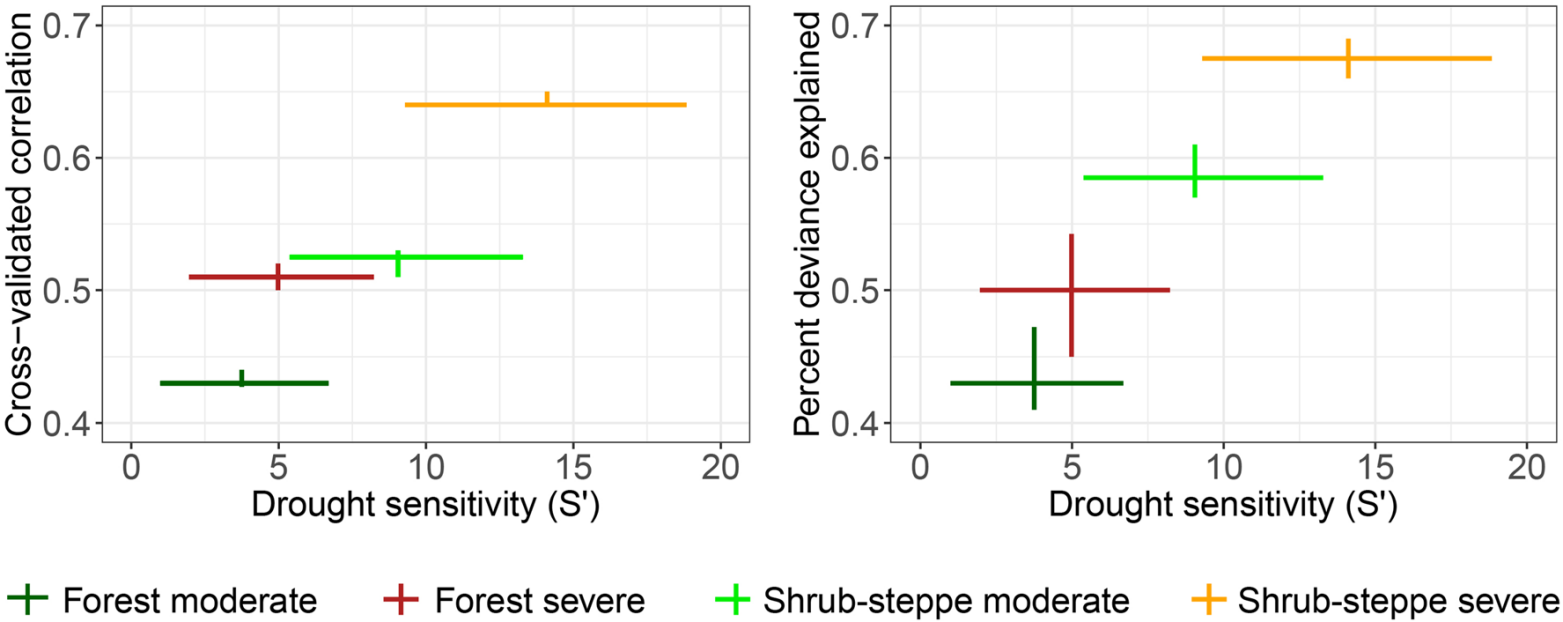
Distribution of drought sensitivity (*S’*) and model fit statistics (cross-validated correlation and percent deviance explained) for the four drought-sensitivity models. Horizontal bars represent the interquartile ranges for *S’* across pixels. Vertical error bars represent interquartile ranges for the model fit statistics across 20 model runs. This figure was created in the R statistical environment^66^.

Models of landscape influences on drought sensitivity produced cross-validated correlation coefficients ranging from 0.43 to 0.64 and explained from 44 to 67% of the deviance in drought sensitivity (medians across 20 model runs; Fig. 6). For both forest and shrub-steppe biomes, these model fit statistics were higher for models of *S’*_*sev*_ than *S’*_*mod*_, indicating that landscape characteristics better explained observed spatial patterns in drought sensitivity when droughts were more intense. Shrub-steppe models outperformed forest models at comparable levels of drought intensity (Fig. 6). Taken together, these patterns indicate that climate and landscape characteristics were better able to explain spatial patterns in drought sensitivity when drought sensitivity was stronger.

## Discussion

Vegetation responses to drought were discernible across two markedly different biomes at a regional scale, with EVI reduced under drought conditions relative to baseline conditions in over 95% of shrub-steppe pixels and 80% of forest pixels. Drought sensitivity in this study likely reflected drought-induced changes to vegetation canopies—such as reductions in leaf area—that can interact with other drought-induced physiological stresses to impact ecosystem productivity^18,23,24,37^. The sensitivity of ecosystem productivity to climate variability has implications for ecosystem services and processes including habitat availability and stability through time, nutrient cycling and carbon storage, watershed hydrology, and diversity of species and plant functional groups^1,30^. In certain cases, productivity reductions from drought may also signal slowdowns in tree growth preceding mortality^38,39^. Such considerations are especially important in regions such as the western United States where drought intensification and climate aridification are projected under climate change^2,40^.

Shrub-steppe ecosystems, which develop under drier climates and have lower biomass than conifer forests, showed stronger drought sensitivity and greater differential response to severe compared to moderate droughts than did forested areas. Previous studies have similarly found greater drought sensitivity in non-forest ecosystems relative to forested ones^28^ and in semi-arid regions compared to humid ones^30^. Water-limited biomes with relatively low gross primary productivity have shown stronger coupling between hydroclimate variation and vegetation greenness^20^, greater productivity decreases in response to extreme precipitation patterns^26^ and heat extremes^24^, and slower recovery after droughts^8^. Thus, at broad spatial scales spanning large climate gradients, our results agree with a general pattern of greater spectral sensitivity to drought in biomes with greater water limitation and lower biomass.

However, variability in drought response can be substantial within biomes and within ecosystems (Fig. 2)^8,20,30^, underscoring the importance of biophysical processes governing soil water availability and plant physiologic responses to water stress^11,12,33^. Within-biome variability can be driven by endogenous factors (i.e., species composition) and by exogenous factors (climate, topographic, soil, and hydrologic characteristics). Ecosystem types explicitly represent species composition and are also shaped by exogenous factors. Ecosystem type added little explanatory power to preliminary models after accounting for exogenous factors (supplementary Fig. S2), suggesting that within-biome variability in drought sensitivity is primarily driven by exogenous factors like climate, topography, and soils. Of these exogenous factors, links between climate and vegetation drought responses have been widely evaluated at regional-to-global scales^8,18–20,28,30^, with fewer large-scale studies accounting for the effects of landscape topography, hydrology, and soil conditions (but see^11^), despite site-scale studies demonstrating the importance of these attributes in shaping vegetation responses to droughts^41,42^.

Evidence from this study suggests that in addition to climate controls, topographic features (e.g., valley bottoms) and soil characteristics that influence water availability to roots (e.g., bulk density and soil AWC) can play important roles in shaping drought sensitivity patterns. Such topographic features and soil characteristics effectively mediate the relationship between meteorological drought (i.e., negative anomalies in precipitation, potentially combined with positive anomalies in evapotranspiration) and its manifestations in hydrologic drought (e.g., negative anomalies in soil water reserves, shallow groundwater, or streamflow) and ecological drought (i.e., impacts on species and ecosystems)^43,44^. For a given regional intensity of meteorological drought, landscape features that boost ecologically available water—such as valley bottoms that concentrate surface runoff and provide access to shallow groundwater—may help ameliorate ecological drought impacts^6,11,31^. Conversely, landscape features that restrict ecologically available water—such as areas with highly compacted soils—may exacerbate ecological drought impacts^45^. Furthermore, links between meteorological drought and ecological drought impacts are shaped by interactions of climate with landscape, geologic, topographic, and soil characteristics, such as in areas where plant community composition is shaped by chronically limited subsurface water storage capacity relative to annual precipitation^12^.

Pacific Northwest forests span large gradients of energy and water availability and showed considerable variation in EVI responses to droughts. Drought sensitivity was generally high in forests with both high climatic water deficit and low AET (indicating chronic water limitation on productivity, Fig. 4), and forest drought sensitivity was highest in dry forests and woodlands such as pinyon-juniper woodlands (Fig. 2). Dry forests are commonly considered drought-tolerant, and their spectral responses to drought may in part reflect adaptations that allow tree species to persist through periods of soil water scarcity by sacrificing short-term productivity^13,15,17,28,46,47^. Indeed, some dry forest types may expand their ranges as climates become drier, displacing more drought-vulnerable vegetation types according to some climate projections^48,49^. However, compared to more mesic forests, dry forests have demonstrated greater drought vulnerability^46^, stronger legacy effects from drought on tree growth^7^, and disproportionate mortality during recent extreme drought in California^50^. Furthermore, because dry forests commonly exist near the absolute climatic limits of forest biomes, they may be particularly vulnerable to drought-triggered transformations to shrublands and grasslands under climate change, especially if wildfires increase in frequency or severity^33,48,51,52^. Thus, our findings underscore the importance of long-term monitoring in dry forest ecosystems—especially along forest ecotones to shrublands and grasslands—as droughts intensify and become hotter^2,4,41,52^.

Our observation of muted spectral response to drought in wetter forests west of the Cascade Mountains (Figs. 2 and 4) does not necessarily imply that these forests are invulnerable to droughts, but rather that EVI does not fully capture the physiological and ecological consequences of drought in these forest types, particularly over the time-scales of this study. Compared to highly water-limited forests, wetter forests have shown reduced coupling between growth rates and interannual variability in climatic moisture^20,47^ and may show more subtle or less immediate canopy changes in response to droughts^20^. However, prolonged drought stress can cause profound impacts on wet forests up to and including mortality, in part because tree species have fewer adaptive responses (e.g., cavitation resistance) to cope with moisture deficits^14^. Such species tend to invest more resources into competing for light (e.g., via height and leaf area) than hydraulic architecture to withstand water stress^47,50^ and may show reduced water-use efficiency under drought^17^. Thus, long-term (i.e., multi-year) droughts and extreme drought conditions (which were not observed in this study) are likely to affect forest growth rates and carbon storage in the wetter forests of the region and to elevate vulnerability to large wildfires and insect outbreaks^34^.

Our results suggest relatively high sensitivity to severe drought for high-elevation forests in Idaho’s Rocky Mountains, whereas subalpine forests of the Cascade Mountains—which are lower in elevation and climatically wetter—showed lower sensitivity (Fig. 2). Although high-elevation energy-limited forests generally have low climatic water deficits, soil water storage and tree root network development may be constrained by shallow, rocky soils. Steep slopes in mountainous areas can facilitate rapid runoff of rainfall and snowmelt, further limiting soil water reserves. High-elevation vegetation is commonly adapted to deep snowpack, creating vulnerability to drought-induced snowpack reductions^42,53^. Relatively high drought sensitivity in Rocky Mountain subalpine forests (i.e., energy-limited environments with low productivity, low AET, and moderately low levels of water limitation) helps explain why forest drought sensitivity was not monotonically related to AET or climatic water deficit (Fig. 4).

Patterns of forest drought sensitivity highlight the importance of considering topographic and soil characteristics that can create heterogeneous soil–water availability within forest ecosystems. For example, effects of meteorological drought may be compounded by compacted soils with limited AWC (Fig. 4)^33^. Conversely, localized forest refugia from drought might be supported by convergent topography and areas of low soil bulk density and high soil AWC^54,55^. Forest drought sensitivity was reduced in convergent areas with high CTI (Fig. 4), such as valley bottoms. Such convergent areas tend to have relatively deep soils and shallow groundwater, receive upslope water subsidies, and may experience cold-air pooling, which can suppress evaporative demand^6,11,31^. In the context of warmer future climates and drought intensification, forested valley bottoms and riparian environments warrant greater examination as potential climate-change refugia^11,56^.

Shrub-steppe drought sensitivity showed complex relationships to geographic patterns of water and energy limitation. In shrub-steppe ecosystems, climatic water deficit generally decreases and AET increases at higher elevations. Somewhat surprisingly, shrub-steppe drought sensitivity increased with elevation, despite greater water availability at higher elevations. Given the strong relative influence of elevation in shrub-steppe models, one explanation is that drought sensitivity was primarily driven by high-elevation, high-productivity areas and that secondarily, for sites at comparable elevations, drought sensitivity increased with increasing water limitation. Dwarf sagebrush showed particularly strong sensitivity to severe droughts. This vegetation community is relatively rare regionally (supplementary Table S1), occurring at cold, high-elevation sites with dry climate and low productivity. Species distribution models project that such cold upland shrublands may lose habitat under future warming^48^. Particularly for severe droughts, shrub-steppe drought sensitivity was strongly related to compacted soils, suggesting that reduced infiltration of rainfall and snowmelt and/or impeded root network development in these areas may have exacerbated drought sensitivity. Unlike in forests, shrub-steppe drought sensitivity was not reduced in areas of high CTI, suggesting that valley bottoms and riparian areas did not provide drought refugia. This may be related to the intermittent nature of many streams in the semi-arid zones of the region, which run dry based on local climate conditions and thus would likely fail to provide subsidies of shallow groundwater during droughts^57^.

Although this analysis revealed several clear patterns of drought sensitivity in relation to climate and landscape drivers, these interpretations require some caveats. Importantly, landscape drivers of drought sensitivity are scale-dependent. The spatial resolution of our analysis (1 km) was appropriate for a regional scale but too coarse to discern microclimatic effects such as those from small streams, shaded hillsides, or localized shallow groundwater^31^. In particular, groundwater depth can be highly variable over small spatial scales and can play an important role in buffering hydrologic responses to climate warming and drought^10^. Additionally, the 1-km EVI scale integrates vegetation responses across a variety of plant functional types. Because shrublands often include an understory component of grasses—including invasive annual grasses such as cheatgrass (*Bromus tectorum*)—drought sensitivity patterns at a 1-km scale likely incorporated spectral effects from both shrub and grassland vegetation. In forests, EVI patterns were likely driven primarily by tree canopy dynamics but may have also incorporated understory vegetation or small non-forested inclusions (e.g., montane meadows) within primarily forested pixels. Future regional studies of drought sensitivity could also include nested, site-level analyses at higher resolutions (e.g., 30 m) to evaluate microclimatic effects and finer-scale drought-sensitivity patterns related to landscape metrics (e.g., topographic shading, water-table depth) that were not evident here^6,11^.

We assessed drought sensitivity using relative rather than absolute declines in EVI under drought conditions. As such, for a given magnitude of drought-induced change in EVI, greater baseline EVI (the denominator, see supplementary material Sect. 1.4) would produce a smaller *relative* EVI change and hence a lower sensitivity value. This may help explain the generally low drought sensitivity values observed in western wet conifer and Mediterranean mixed conifer forests west of the Cascade Mountains, which have relatively high baseline EVI (Fig. 2). Absolute declines in EVI under drought conditions could potentially be used as a complementary metric to assess drought sensitivity—along with the relative declines presented here—although such an absolute metric would tend to understate drought effects in strongly water-limited ecosystems with extremely low baseline EVI (e.g., shrub-steppe ecosystems and pinyon-juniper woodlands; Fig. 2), because these ecosystems have “so little to lose” in terms of EVI decreases when droughts occur.

Although vegetation indices are reliable indicators of plant structural and physiological properties (e.g., ANPP and leaf-area index^18,23,24,37,41^) and have enabled broad-scale synoptic assessments of vegetation responses to climate variability, remote-sensing analyses are inherently constrained by a common set of limitations. Spectral change is an imperfect indicator of physiological or ecological responses to drought^28^, in part because physiological responses such as stomatal closure that minimally affect leaf area may produce no discernible spectral change. Conversely, some drought-tolerance adaptations such as leaf shedding and changes to leaf orientation may produce spectral changes without any long-term adverse impacts to plant fitness^13^, such that drought-tolerant vegetation types may show strong drought sensitivity in remote-sensing analysis^28^. Indeed, productivity reductions from drought discernible by remote sensing do not necessarily imply drought-induced mortality or plant community vulnerability to the long-term negative effects of drought. Furthermore, drought-induced reductions in plant fitness—including morbidity effects that cannot be detected by remote sensing—may contribute to delayed mortality with varying lag times, including through drought-related disturbances such as wildfires and insect outbreaks^7,27,38,39^. Thus, the drought sensitivity patterns explored here and in other remote-sensing-based studies represent only one facet of holistic drought-vulnerability analysis, which also requires field-based tracking of mortality events, long-term monitoring of population demographics, species composition, physiological traits, and drought projections under climate change.

## Conclusions

Improved understanding of broad-scale drivers of drought sensitivity is critical for predicting where and when ecosystems will experience substantial declines in productivity under future droughts and potentially where to expect transitions in ecosystem types. Droughts are anticipated to intensify under climate change and recent severe drought in the Pacific Northwest may not be as anomalous in years to come^2,35^. Our findings of elevated drought sensitivity in dry forests, Rocky Mountain subalpine forests, and cold upland sagebrush communities suggest these ecosystems may warrant long-term monitoring and plot-scale studies of plant physiological responses to drought. We identified landscape contexts with reduced drought sensitivity—such as forested valley bottoms and forests with low soil bulk density and high soil AWC—that may require further investigation as potential drought refugia^11,31,33,54–56^.

The sensitivity of ecosystem productivity to drought—investigated in previous studies and here using EVI as an indicator of ANPP^20,23,28^—may have complex relationships to long-term drought vulnerability, susceptibility to mortality events, and ecosystem transitions under climate change. Remotely sensed indicators of drought sensitivity integrate the effects of biological characteristics (e.g., drought-resistance strategies) and physical landscape characteristics (e.g., heterogeneous soil water availability), and climate change will affect these drivers to different degrees over different time-scales^29^. Future studies integrating drought-sensitivity analysis with long-term post-drought monitoring may help elucidate the relationships between vegetation productivity declines and residual impacts from droughts, e.g., reduced reproductive success or delayed mortality^1,38^. Furthermore, because ecosystems face interacting threats (e.g., invasive species and insect outbreaks) and shifting disturbance regimes (e.g., longer fire seasons), assessing drought vulnerability in concert with these changing processes can help guide landscape-scale management under climate change.

## Methods

### Study area

We assessed drought sensitivity in conifer forest and shrub-steppe biomes of the Pacific Northwest of the USA (Oregon, Washington, and Idaho). Elevation in this region ranges from sea level to approximately 4,300 m. Mean annual temperatures (1981–2010 averages) range from − 7.0 °C on mountain tops to 13.3 °C in lowland and coastal areas^58^. Mean annual precipitation (MAP) ranges from approximately 20 to 650 cm per year. In the semi-arid regions east of the Cascade Mountains where MAP < 40 cm, natural landcover includes shrub-steppe ecosystems dominated by sagebrush (*Artemisia* species). Natural landcover in areas west of the Cascades or at elevations above 1200–1400 m is generally conifer forest.

### Remote sensing, landcover, and climate data

We quantified vegetation greenness using MODIS EVI from EarthData Search^59^ at 1-km resolution for June through August, 2000 through 2016 (part 1 in supplementary material). We used only EVI pixels representing natural landcover based on the U.S. National Vegetation Classification^60^—seven forest ecosystems and three shrub-steppe ecosystems (supplementary Table S1)—and excluded pixels that were influenced by disturbances such as fires or insect outbreaks or that showed long-term EVI trends (part 1 in supplementary material; supplementary Fig. S1).

We assessed drought conditions using the standardized precipitation evapotranspiration index (SPEI), which incorporates precipitation and temperature effects to represent meteorological drought conditions across time and space^61^. Because biomes may differ in the time-scales over which they respond to drought^20^, we represented drought conditions over three time-scales using SPEI calculated with 3-, 6-, and 12-month antecedent conditions, representing drought conditions for summer, winter through summer, and the entire previous year, respectively (part 1 in supplementary material). We defined baseline climate conditions as − 1 < SPEI < 1, moderate drought as − 1.5 < SPEI ≤ -1, and severe drought as SPEI ≤ − 1.5, following similar SPEI thresholds from previous studies^2,3^. Cumulative drought exposure for each pixel was calculated using SPEI from 1990 through 2016 (part 1 in supplementary material).

### Drought sensitivity

We represented sensitivity to moderate and severe drought (*S’*_*mod*_ and *S’*_*sev*_, respectively) as the percent decrease in EVI under drought conditions relative to baseline (i.e., non-drought, non-pluvial) conditions. We first calculated drought sensitivity for each SPEI timeframe (3-, 6-, and 12-months) and then calculated *S’* as the maximum sensitivity across timeframes (part 1 in supplementary material). Although some ecologically important manifestations of drought sensitivity (e.g., below-ground physiological changes, reduced defense capacity against pests and pathogens) are not directly captured in EVI variability, EVI is closely linked to ANPP^18,23,24^. Therefore, this metric of drought sensitivity represents the degree to which vegetation canopies exhibit spectral changes in response to drought and may indicate drought-induced productivity reductions. This drought-sensitivity analysis likely represents a conservative assessment of regional drought impacts because we did not seek to explicitly map vegetation mortality and we excluded areas affected by fires, insect outbreaks, and other disturbances. In some of these excluded areas, droughts could have caused vegetation mortality directly or may have contributed to or exacerbated fire and insect disturbances (part 1 in supplementary material)^1,41,62^.

### Models of landscape characteristics influencing drought sensitivity

We assessed drought sensitivity by ecosystem type and used boosted regression tree (BRT) models to explore influences on drought sensitivity of landscape characteristics including climate, topography, soil, and hydrology (Table 1). BRT models are a machine-learning technique based on classification and regression tree models, which repeatedly split values of a response variable into more homogenous groups using combinations of predictor variables^63^. BRTs are effective in modeling complex ecological systems because they accommodate continuous and categorical predictors, missing data, and outliers, do not require prior data transformation, and capture complex nonlinear relationships^64^.

**Table 1:** Landscape characteristics used in evaluating drought-sensitivity patterns.

Landscape characteristic	Units	Source	Native resolution	Pre-processing prior to use in boosted regression tree (BRT) models	Abbreviation used in BRT models
Level III ecoregion	Code	Omernik ecoregions^68^	Vector polygons	Rasterized at 1 km to match the grid of *S’* values	Not used
Landcover and ecosystem type (level 5 macrogroup categories, supplementary Table S1)	Code	U.S. National Vegetation Classification^60^ mapped by the National Gap Analysis Program^69^	30 m	Landcover for each 1-km pixel was categorized as the dominant (majority) category of the 30-m landcover within the pixel. Only pixels with one of the ecosystem types in supplementary Table S1 were used for analysis	Ecosystem^†^
Elevation	m	GTOPO30^70^	30 arc-s (~ 800 m)	Resampled using bilinear interpolation to match the 1-km grid of *S’* values	Elev
Actual evapotranspiration (1980–2009)	mm	AdaptWest^71^	30 arc-s	Same resampling as for elevation	AET
Climatic water deficit (1980–2009)	mm	AdaptWest^71^	30 arc-s	Same resampling as for elevation	Deficit
Soil available water capacity	Volumetric fraction	SoilGrids^72^	1 km	Same resampling as for elevation	Soil_AWC
Soil bulk density	kg per m^3^	SoilGrids^72^	1 km	Same resampling as for elevation	Soil_BD
Groundwater-table depth	m	Fan et al.^73^	1 km	Same resampling as for elevation	WTD
Compound topographic index (CTI)	Unitless	Buttrick et al.^36^	30 m	Averaged within each 1-km pixel	CTI
Topographic heat-load index (HLI)	Unitless	Buttrick et al.^36^	30 m	Averaged within each 1-km pixel to produce 1-km HLI	HLI^†^
				Density of topographic shading = percent of each 1-km pixel with HLI ≤ 0.6	Shade_dens^†^
Drought exposure	Percent	This study	1 km	See supplementary methods	Drought_exp

Notes: Pixels were assigned to forest or shrub-steppe models based on landcover and ecoregion. Landscape characteristics marked with † were removed from final models based on relative-influence values in preliminary models (see supplementary methods).

We constructed four independent models for each combination of drought intensity level (*S’*_*mod*_ and *S’*_*sev*_) and biome (forest and shrub-steppe). Parameterization of BRTs was conducted using the gbm.step function in the R package dismo^65^, with tree complexity of five and a bag fraction of 0.5 (part 1 in supplementary material). For each model, we performed 20 bootstrapped BRT model runs using a random sample of 10,000 pixels and averaged the results. We assessed model fit based on percent deviance of the response variable (drought sensitivity) explained by the model and by the cross-validated correlation coefficient based on tenfold cross validation in the gbm.step function. We evaluated the roles of landscape variables in shaping spatial patterns of drought sensitivity within each biome based on relative influence (i.e., the importance of each predictor in explaining drought sensitivity patterns) and the shapes of partial-dependence plots, which depict drought sensitivity as a function of each landscape characteristic while accounting for the average effects of all other predictors in the model^64^.

We first constructed preliminary BRT models with 11 predictors: elevation, climatic water deficit, AET, soil bulk density, soil AWC, groundwater-table depth, CTI, topographic heat-load index (HLI), density of topographic shading, drought exposure, and ecosystem type (Table 1). Correlations between predictors were generally weak to moderate (supplementary Table S2). Three predictors (HLI, density of topographic shading, and ecosystem type) had low relative influence in preliminary models (supplementary Fig. S2) and were removed to create more parsimonious final models. All data processing and modeling were conducted in the R statistical environment^66^. Geospatial data, metadata, and data-processing scripts are available in a U.S. Geological Survey data release^67^.

## Supplementary information

Supplementary Information.

## Data Availability

All data and code (R processing scripts) used in this analysis are available in a U.S. Geological Survey data release^67^.
