# Supplementary material for: Topographic, soil, and climate drivers of drought sensitivity in forests and shrublands of the Pacific Northwest, USA
Source: Sci Rep. 2020 Oct 28;10:18486. doi: 10.1038/s41598-020-75273-5 (PMC7595234; doi:10.1038/s41598-020-75273-5)
Supplement: Supplementary file 1 — Supplementary Information. [file 41598_2020_75273_MOESM1_ESM.docx]

**Topographic, soil, and climate drivers of drought sensitivity in forests and shrublands of the Pacific Northwest, USA**

Cartwright J. M., Littlefield C. E., Michalak J. L., Lawler J. J., and Dobrowski S. Z.

**Supplementary materials**

**Part 1. Supplementary methods**

**1.1. Enhanced Vegetation Index**

We quantified vegetation greenness using remotely sensed enhanced vegetation index (EVI) from the Moderate-resolution Imaging Spectroradiometer (MODIS). Monthly composite EVI datasets at 1-km resolution (the MOD13A3 product) were obtained from EarthData Search^1^ (<https://search.earthdata.nasa.gov/search>) for summer (June, July, and August) of 2000 through 2016. Three tiles covering the study area (h08v04, h09v04, and h10v04) were mosaicked to produce a single EVI grid for each monthly time step. EVI pixels affected by clouds, snow, and ice were removed using the pixel reliability files included with the EVI datasets. All EVI processing, spatial analyses, and modeling were conducted in the R statistical environment^2^. Geospatial data, metadata, and data-processing scripts in the R statistical language are available in a U.S. Geological Survey data release^3^.

**1.2. Landcover and disturbance screens**

We assessed drought sensitivity in minimally disturbed (‘intact’) conifer forest and shrub-steppe ecosystems (fig. S1). Using mapped landcover categories^4,5^, we identified seven forest ecosystem types and three shrub-steppe ecosystem types that each covered at least 1% of the study area (table S1). We removed all other landcover types from analysis, including agriculture, open water, and developed areas. We removed a small number (< 2 %) of shrub-steppe pixels located west of the Cascade Mountains because these small, isolated shrub patches are environmentally different from the semi-arid shrub-steppe ecosystems east of the Cascades.

To exclude the influence of disturbance interactions, we removed pixels from analysis that had been affected by recent fires or insect outbreaks. We calculated the percentage of each 1-km pixel that was burned from 1984 to 2015 by summing rasterized versions of annual fire perimeters from the Monitoring Trends in Burn Severity dataset^6^, and we removed pixels with > 10% area burned. We also removed pixels with > 1 tree per hectare of cumulative insect mortality from 1991 to 2016. Annual aerial survey polygons quantifying insect mortality^7^ were rasterized at 100-m resolution and summed across years to derive cumulative mortality. Cumulative mortality for each 1-km pixel was calculated as the mean of 100-m resolution cumulative mortality within the pixel.

To screen out pixels affected by other forms of disturbance not captured in the above datasets (e.g., timber harvest or storm damage), we examined each pixel’s Pearson correlation between year and baseline EVI (i.e., average EVI under non-drought, non-pluvial conditions, as defined in the section ‘Drought sensitivity’). Cells with significant (p < 0.05) increases or decreases in baseline EVI over time were excluded from drought-sensitivity analysis because their changes in EVI may have been influenced by factors other than variability in climatic water inputs^8^.


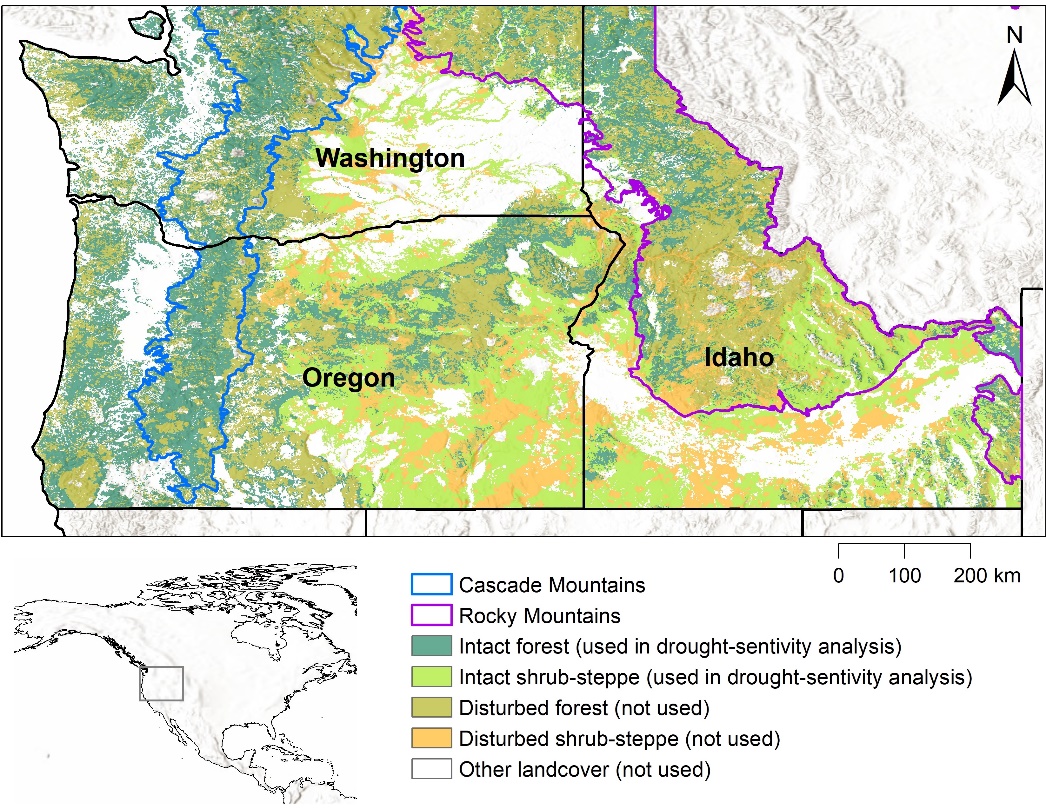
We acknowledge that some cells with significantly negative EVI trends may represent areas of drought-induced mortality^9,10^ and that some cells affected by fires and insect outbreaks may represent areas where droughts contributed to or exacerbated these disturbances^11^. However, causal attribution of these effects to droughts is difficult^10^, and we could not conclude with certainty that vegetation changes in these disturbed cells were drought-induced as mortality due to fires, insect outbreaks, and other disturbances also occurs in the absence of drought. Thus, drought-sensitivity patterns considered in this study represent conservative assessments of regional drought effects, as droughts also likely contributed to vegetation mortality through complex disturbance interactions in some areas excluded from our analysis.

**Figure S1.** Drought sensitivity was assessed in minimally disturbed (‘intact’) forest and shrub-steppe ecosystems in the Pacific Northwest of the United States. Forest and shrub-steppe pixels that were affected by recent fire or insect outbreaks and those that exhibited significant trends in baseline EVI over time (‘disturbed’) were excluded from analysis, using landcover and disturbance data obtained from^5–7^. The Cascade Mountains boundary represents the Cascades and North Cascades level 3 ecoregions within the study area^12^. The Rocky Mountains boundary represents the Northern Rockies, Middle Rockies, and Idaho Batholith level 3 ecoregions within the study area^12^. Data processing was performed in the R statistical environment^2^. Maps were created using Esri ArcGIS Desktop v.10.4.1^13^. Basemap courtesy of: Esri, USGS, NGA, NASA, CGIAR, N Robinson, NCEAS, NLS, OS, NMA, Geodatastyrelsen, Rijkswaterstaat, GSA, Geoland, FEMA, Intermap and the GIS user community.

| **Table S1**. Ecosystem types used in drought sensitivity analysis | | |
| --- | --- | --- |
| Biome | Level 5 macrogroup name (code)  *Ecosystem short name (abbreviation)* | Percent of study area |
| Forest | Northern Rocky Mountain Lower Montane and Foothill Forest (17)  *Rocky Mountain mixed conifer (RMMC)* | 17.6 |
| Forest | Californian-Vancouverian Foothill and Valley Forest and Woodland (19)  *Mediterranean pine-oak woodland (MPOW)* | 1.7 |
| Forest | Rocky Mountain Subalpine and High Montane Conifer Forest (20)  *Rocky Mountain subalpine (RMS)* | 7.3 |
| Forest | Southern Vancouverian Montane and Foothill Forest (23)  *Mediterranean mixed conifer (MMC)* | 2.0 |
| Forest | Vancouverian Lowland and Montane Rainforest (24)  *Western wet conifer (WWC)* | 11.5 |
| Forest | Vancouverian Subalpine Forest (25)  *Cascade subalpine (CS)* | 1.4 |
| Forest | Intermountain Singleleaf Pinyon - Western Juniper Woodland (26)  *Pinyon-juniper (PJ)* | 2.2 |
| Shrub-steppe | Northern Rocky Mountain-Vancouverian Montane and Foothill Grassland and Shrubland (48)  *Rocky Mountain shrubland and grassland (RMSG)* | 3.9 |
| Shrub-steppe | Great Basin and Intermountain Tall Sagebrush Shrubland and Steppe (169)  *Tall sagebrush (TS)* | 24.2 |
| Shrub-steppe | Great Basin and Intermountain Dwarf Sage Shrubland and Steppe (170)  *Dwarf sagebrush (DS)* | 1.9 |
| Notes: level 5 macrogroup names and codes (in parentheses) are from the U.S. National Vegetation Classification^4^. Shorter names for ecosystem types used in this analysis, along with abbreviations used in fig. 2 in the main text, are in italics. | | |

**1.3. Quantifying drought**

We assessed drought conditions using the standardized precipitation evapotranspiration index (SPEI)^14^. Because biomes differ in the time-scales over which they respond to drought^15,16^, SPEI’s ability to integrate across multiple periods of antecedent conditions is advantageous to capture different seasonal and cumulative effects^14^.

We represented drought conditions over three time-scales (*T*) using SPEI calculated with 3-, 6-, and 12-month antecedent conditions (SPEI_03_, SPEI_06_, and SPEI_12_, respectively) obtained from the West Wide Drought Tracker^17^. All gridded SPEI datasets were resampled using bilinear interpolation to match the EVI grids. Because we restricted our analysis to summer (June, July, and August), SPEI_03_ represented drought conditions in the growing season, SPEI_06_ also included winter and early spring drought effects, and SPEI_12_ incorporated the entire previous year’s moisture conditions. We defined baseline (non-drought, non-pluvial) climate conditions for each time-scale as -1 < SPEI < 1 and examined drought sensitivity separately for two drought intensity levels (*L*): moderate drought (-1.5 < SPEI ≤ -1) and severe drought (SPEI ≤ -1.5), following similar SPEI breakpoints to define drought intensity^18–20^.

We quantified summer drought exposure from 1990 through 2016 for each pixel (*P*) and each intensity level (*L*) as the percentage of summer months during these years that *P* experienced drought conditions based on any time-scale (*T*) (i.e. SPEI_03_, SPEI_06_, or SPEI_12_). Because the EVI observations began in 2000, we included drought observations back to 1990 to allow for lag effects on drought response, i.e., influences from previous drought exposure on drought sensitivity. At each monthly time-step, *P* was considered to be in moderate drought if SPEI for any *T* was between -1.5 and -1, to be in severe drought if SPEI for any *T* was less than -1.5, and to be experiencing ‘any drought’ (either moderate or severe) if SPEI for any *T* was less than -1.

**1.4. Drought sensitivity**

We developed an index of drought sensitivity (*S’*) to represent the percent decrease in EVI under drought conditions relative to baseline conditions. First, for each pixel (*P*) we identified the EVI observations associated with baseline climate conditions across all time-scales (*T*); i.e., EVI observations for which SPEI_03_, SPEI_06_, and SPEI_12_ all indicated non-drought, non-pluvial conditions. If pixel *P* had ≥ 3 such baseline-associated EVI observations, we calculated baseline EVI*_P_* as the mean across these EVI observations. Similarly, we calculated drought EVI*_P_* for each combination of *T* and *L* as the mean across drought-associated EVI observations for pixel *P*.

We calculated drought sensitivity (*S*) for each combination of *T* and *L*:

*S_P,T,L_* = [ (baseline EVI*_P_* – drought EVI*_P,T,L_*) / baseline EVI*_P_* ] x 100

We calculated *S’* for each drought-intensity level *L* as the maximum of *S* across time scales (*T*):

*S’_P, mod_* = max (*S_P, 03, mod_*, *S_P, 06, mod_*, *S_P, 12, mod_*)

*S’_P, sev_* = max (*S_P, 03, sev_*, *S_P, 06, sev_*, *S_P, 12, sev_*)

where *mod* and *sev* represent moderate and severe drought-intensity levels, respectively. A small number of pixels had < 3 baseline-associated EVI observations and/or had no drought-associated EVI observations for some combinations of *T* and *L*, such that *S’* could not be calculated. This affected 1.5% and 9.7% of forest pixels and 0.4% and 4.4% of shrub-steppe pixels, for *S’_mod_* and *S’_sev_*, respectively.

**1.5. Boosted regression tree models**

We examined relationships between *S*’ and landscape characteristics representing climate, topography, soil, and hydrology (table 1 in main text). For the most part, landscape variables were weakly to moderately correlated with each other (table S2).

We systematically evaluated multiple parameterizations of BRTs using the gbm.step function in the R package dismo^21^ to optimize model skill and computing efficiency^22^. Tree complexity (TC) was set to five and the bag fraction was 0.5. After evaluating learning rates of 0.005, 0.01, and 0.02, which reflect the contribution of each tree to the growing model, we selected a learning rate of 0.01 to optimize efficiency and avoid overfitting^22,23^. We completed 20 BRT model runs for each of the four drought sensitivity types, using a random sample of 10,000 pixels for each model run (7-9% of available pixels). This bootstrapping approach allowed us to evaluate variability across model runs to inform our level of confidence in the emergent relationships between predictors and drought sensitivity.

Preliminary BRT models had 11 explanatory variables: elevation, climatic water deficit, actual evapotranspiration, soil bulk density, soil available water content (AWC), groundwater-table depth (WTD), compound topographic index (CTI), topographic heat-load index (HLI), density of topographic shading, drought exposure, and ecosystem type (table 1 in main text). In preliminary BRT models, HLI, density of topographic shading, and ecosystem type showed consistently low relative influence across models (fig. S2), so these predictors were removed to create more parsimonious final models. We retained drought exposure in the final BRT models despite generally low relative influence in the preliminary models to explore the possible effects of previous drought exposure on drought sensitivity.

| **Table S2**. Correlations among landscape characteristics used in preliminary BRT models | | | | | | | | | |
| --- | --- | --- | --- | --- | --- | --- | --- | --- | --- |
|  | AET | Deficit | Elev | Soil_AWC | Soil_BD | CTI | HLI | Shade_dens | WTD |
| AET |  | **-0.79** | 0.28 | -0.03 | -0.49 | -0.46 | 0.11 | 0.36 | -0.06 |
| Deficit | -0.34 |  | -0.41 | -0.11 | **0.59** | 0.40 | -0.03 | -0.31 | 0.13 |
| Elev | **-0.63** | 0.18 |  | 0.04 | -0.40 | -0.05 | 0.30 | -0.08 | -0.03 |
| Soil_AWC | -0.18 | **-0.50** | 0.36 |  | -0.33 | 0.06 | -0.12 | -0.04 | -0.26 |
| Soil_BD | -0.20 | **0.77** | -0.03 | **-0.71** |  | 0.28 | -0.08 | -0.24 | 0.13 |
| CTI | -0.06 | 0.26 | -0.10 | -0.20 | 0.42 |  | -0.08 | **-0.79** | -0.14 |
| HLI |  | 0.14 | 0.11 | -0.08 | 0.02 | -0.07 |  | -0.29 | 0.13 |
| Shade_dens |  | -0.23 | 0.05 | 0.21 | -0.29 | **-0.58** | **-0.63** |  | 0.04 |
| WTD | -0.30 | 0.13 | 0.34 | 0.09 |  | -0.18 | 0.10 | 0.09 |  |
| Notes: values are statistically significant (p < 0.05) Spearman correlation coefficients using random samples of 10,000 pixels; insignificant correlations are blank. Green and yellow cells represent correlations for samples of forest and shrub-steppe pixels, respectively. Correlations with absolute values ≥ 0.5 are bolded. Variables are defined in table 1 of the main text. | | | | | | | | | |

**
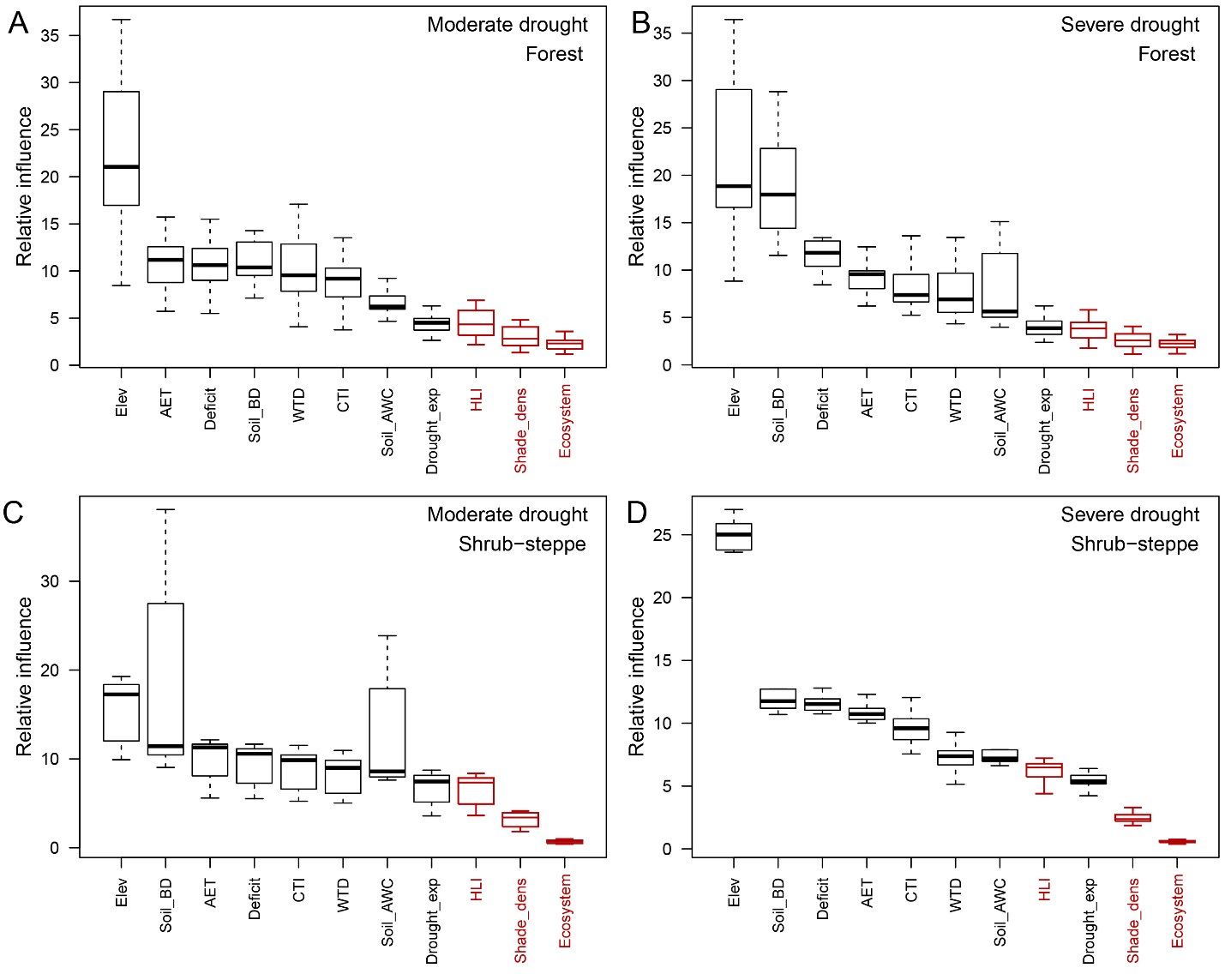
**

**Figure S2.** Relative influence distributions for preliminary boosted-regression tree (BRT) models of (a) *S’_mod_* in forest, (b) *S’_sev_* in forest, (c) *S’_mod_* in shrub-steppe, and (d) *S’_sev_* in shrub-steppe biomes. Boxplots represent distributions of relative influence for predictor variables (defined in table 1 of the main text) across 20 independent model runs, showing interquartile ranges and medians (boxes) and minimum and maximum values (whiskers). Variables colored red were dropped from the final BRT models due to low relative influence. This figure was created in the R statistical environment^2^.

**Part 2. Supplementary modeling results**

**2.1. Partial-dependence plots across bootstrapped model runs**

For each combination of drought-intensity level (moderate and severe) and biome (forest and shrub-steppe), partial-dependence plots were overlaid for each of the 20 bootstrapped BRT model runs (figs. S3 through S6). The smoothed average across model runs (red lines in figs. S3 through S6) are presented in the partial-dependence plots in the main text.

Although BRT models produced partial-dependence relationships across the full range of each predictor variable, some partial-dependence plots showed a high degree of variability across the 20 model runs at the highest and lowest values for some predictors (figs. S3 through S6). At the tails of the distributions of predictors, stochastic instability across model runs can result from relatively few observations (pixels). Thus, our interpretation of partial-dependence plots to discern landscape controls on drought sensitivity was focused on the regions between the 5th and 95th percentiles of each predictor (vertical dashed lines in partial dependence plots).


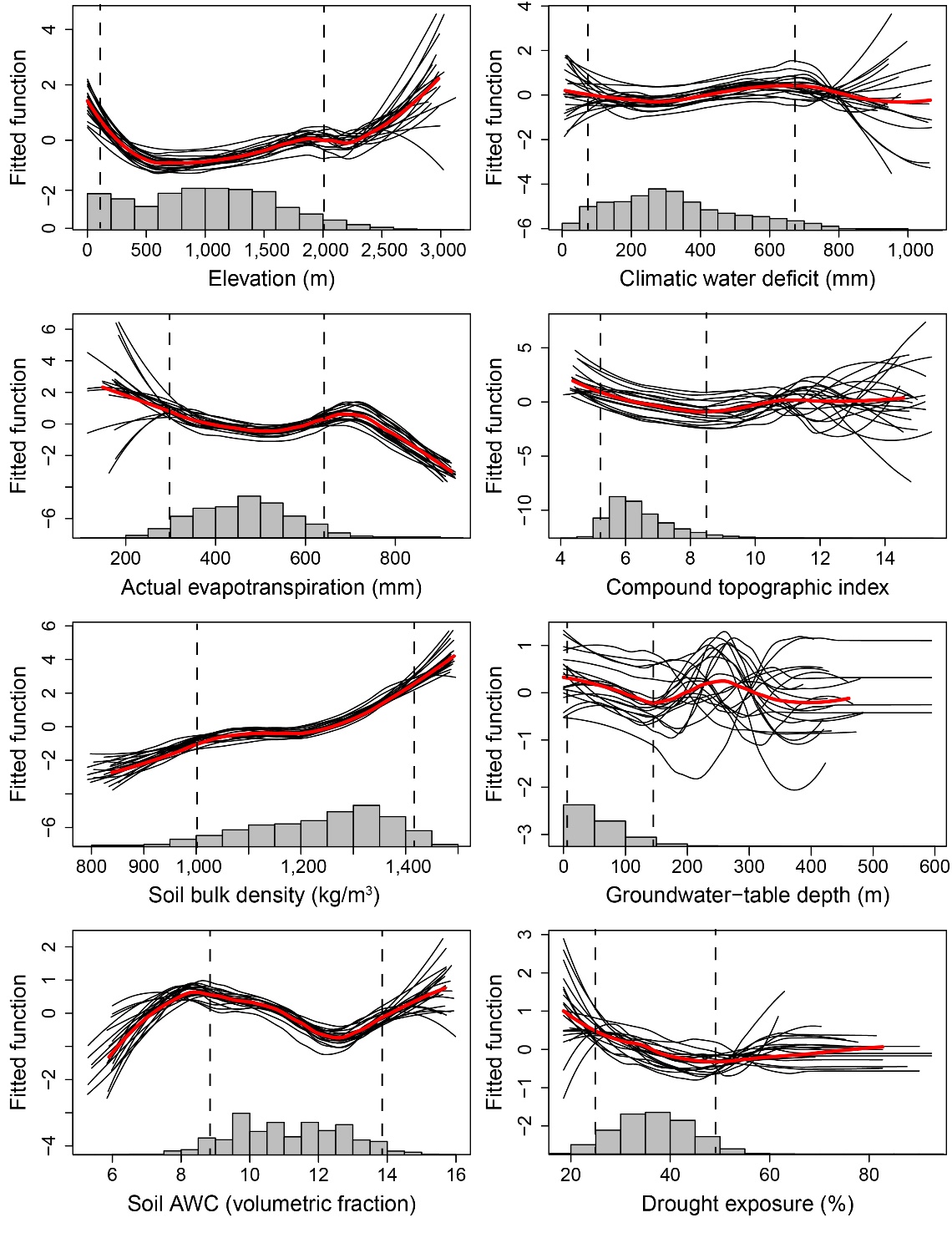


**Figure S3.** Partial-dependence plots showing marginal influence on forest sensitivity to moderate drought of boosted-regression tree model predictors (defined in Table 1 in the main text). Each plot includes 20 black lines representing individual model runs using random subsets of 10,000 pixels and a bold red line indicating a smoothed average across model runs. Histograms show distributions of predictors across all forest pixels used in modeling. Interpretation of partial-dependence plots uses the regions between the 5th and 95th percentiles of each predictor (vertical dashed lines). This figure was created in the R statistical environment^2^.


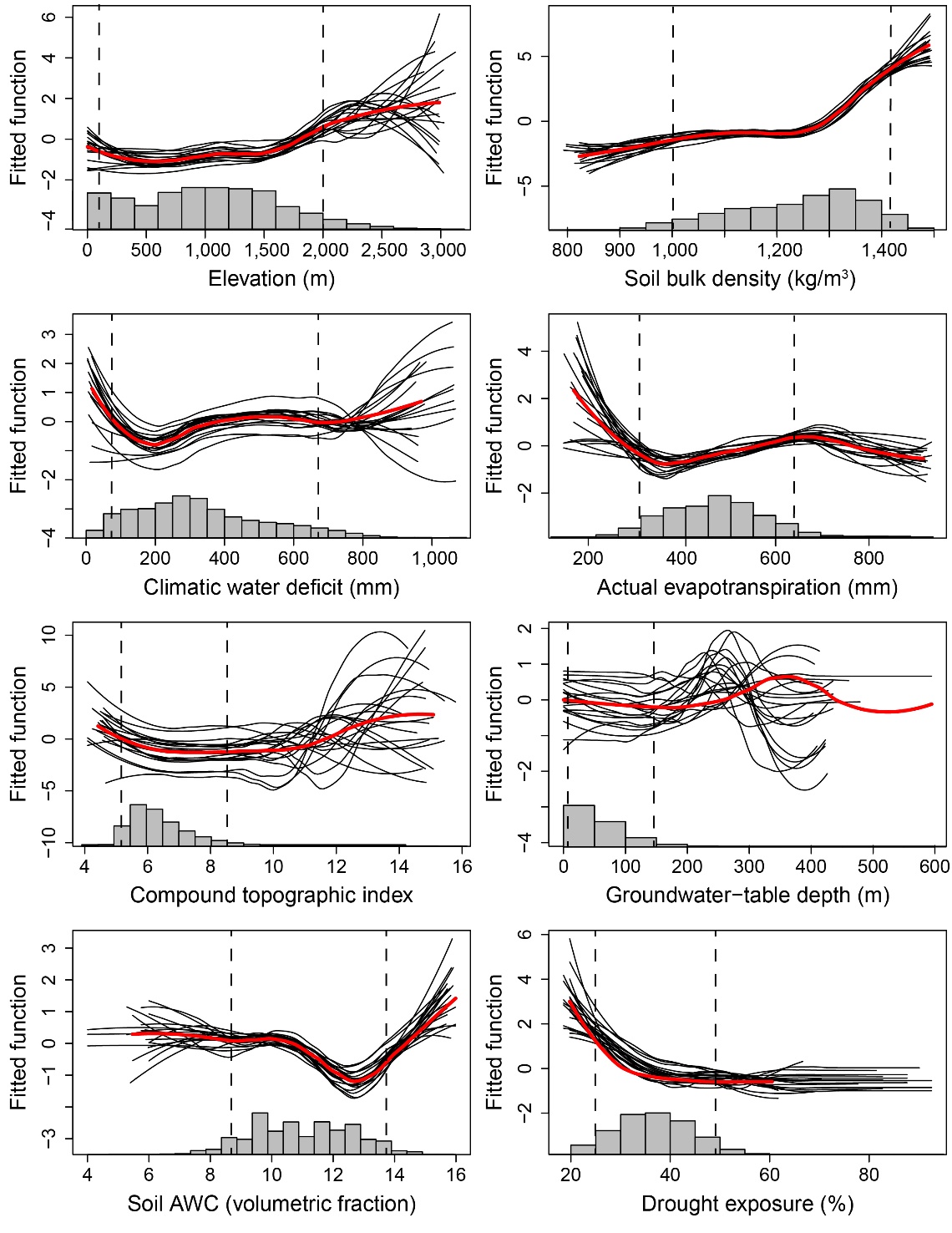


**Figure S4.** Partial-dependence plots showing marginal influence on forest sensitivity to severe drought of boosted-regression tree model predictors (defined in Table 1 in the main text). Each plot includes 20 black lines representing individual model runs using random subsets of 10,000 pixels and a bold red line indicating a smoothed average across model runs. Histograms show distributions of predictors across all forest pixels used in modeling. Interpretation of partial-dependence plots uses the regions between the 5th and 95th percentiles of each predictor (vertical dashed lines). This figure was created in the R statistical environment^2^.


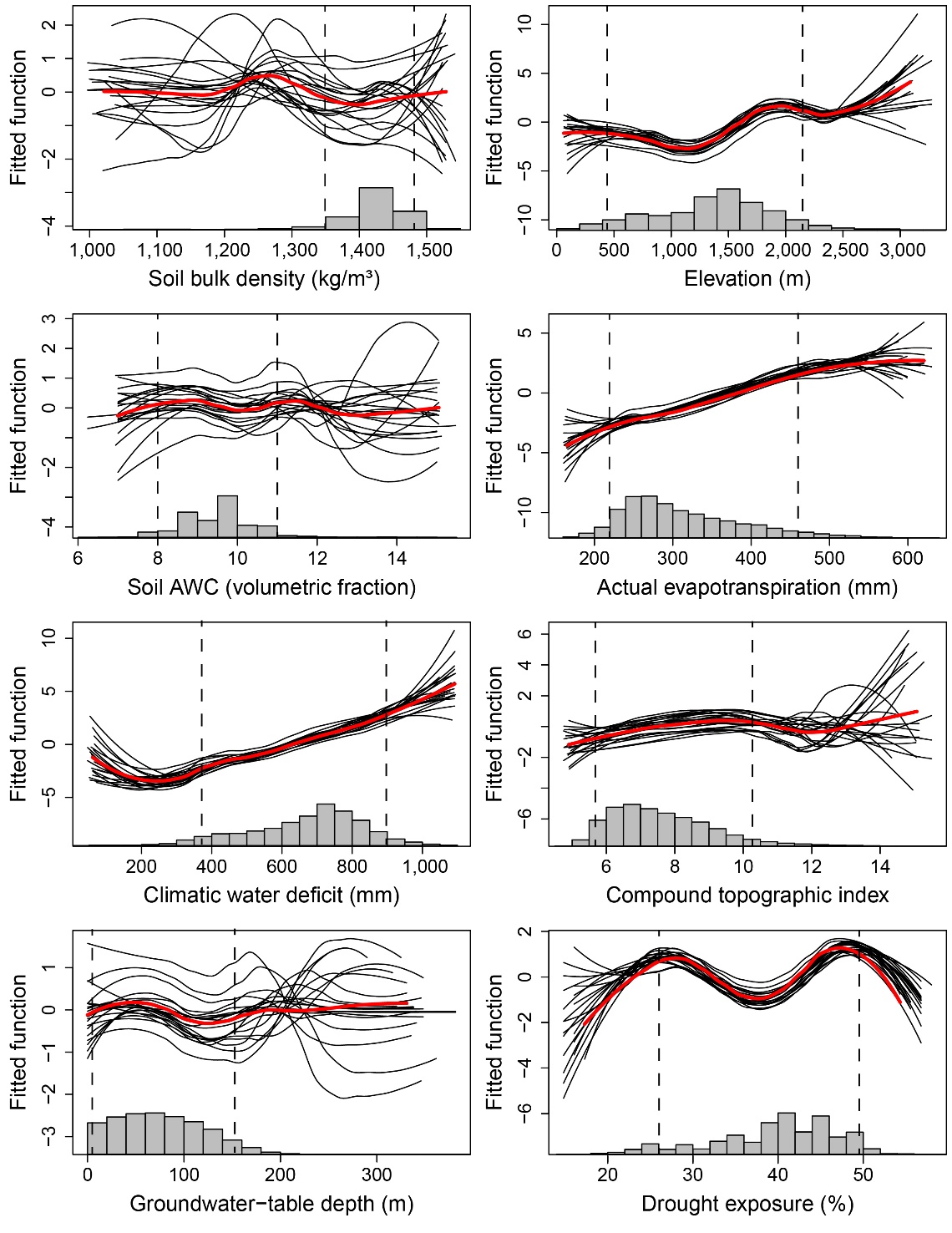


**Figure S5.** Partial-dependence plots showing marginal influence on shrub-steppe sensitivity to moderate drought of boosted-regression tree model predictors (defined in Table 1 in the main text). Each plot includes 20 black lines representing individual model runs using random subsets of 10,000 pixels and a bold red line indicating a smoothed average across model runs. Histograms show distributions of predictors across all shrub-steppe pixels used in modeling. Interpretation of partial-dependence plots uses the regions between the 5th and 95th percentiles of each predictor (vertical dashed lines). This figure was created in the R statistical environment^2^.


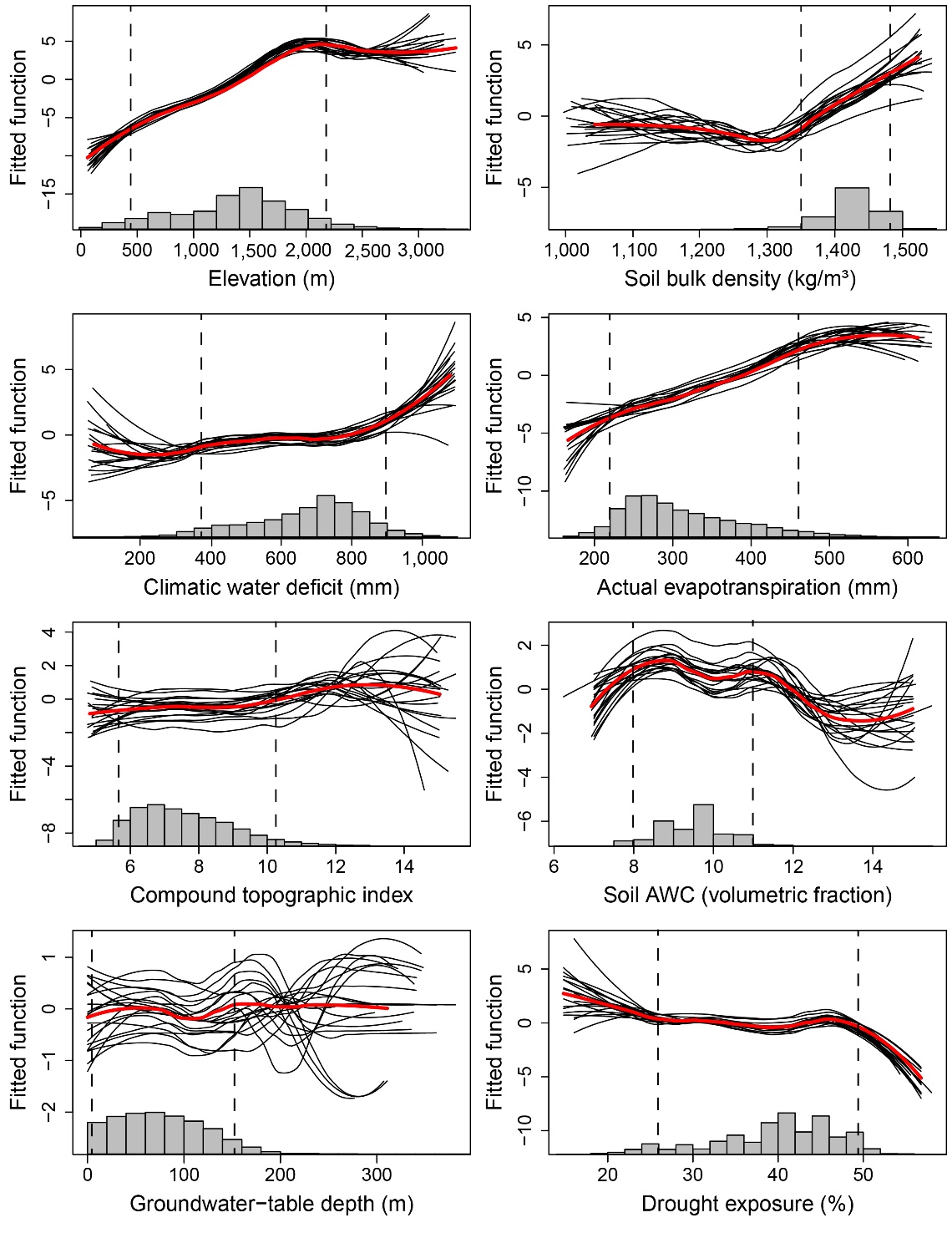


**Figure S6.** Partial-dependence plots showing marginal influence on shrub-steppe sensitivity to severe drought of boosted-regression tree model predictors (defined in Table 1 in the main text). Each plot includes 20 black lines representing individual model runs using random subsets of 10,000 pixels and a bold red line indicating a smoothed average across model runs. Histograms show distributions of predictors across all shrub-steppe pixels used in modeling. Interpretation of partial-dependence plots uses the regions between the 5th and 95th percentiles of each predictor (vertical dashed lines). This figure was created in the R statistical environment^2^.

**2.2. Groundwater-table depth patterns**

Partial-dependence plots showed particularly high variability across model runs for the groundwater-table depth predictor variable and did not show compelling evidence for reduced drought sensitivity in areas of shallow groundwater (figs. S3 through S6). Groundwater depths for forest and shrub-steppe pixels were predominantly mapped as greater than 20 m below the land surface (84% and 89%, respectively), depths that would be inaccessible to most plants and which likely underrepresented localized shallow groundwater availability at sub-kilometer scales (e.g., along floodplains and riparian zones). In the absence of a regionally comprehensive, high-resolution dataset representing groundwater depth, we used estimates from a global-scale model^24^, however, our results suggest that groundwater influences on drought sensitivity were not well captured by using this dataset. Groundwater depth can be highly variable over small spatial scales (<<1 km) due to underlying geologic features that are difficult to map at high resolutions and can change seasonally, in response to climate variability, and from groundwater withdrawals^24^. Groundwater is known to play an important role in buffering hydrologic responses to climate warming and drought^25–27^, e.g., as discharge in springs and seeps and as baseflow in streams influencing riparian areas, however, these microenvironments were not captured at the scale of our analysis. To address these issues, future studies of drought sensitivity at regional scales may require a higher resolution analysis with locally validated groundwater depth estimates to check for microscale effects on drought sensitivity, particularly with respect to localized areas of shallow groundwater availability.

**Acknowledgements**

Any use of trade, firm, or product names is for descriptive purposes only and does not imply endorsement by the U.S. Government.

**References**

1. National Aeronautics and Space Administration (NASA). EarthData Search. (2020). Available at: https://search.earthdata.nasa.gov/search. (Accessed: 8th January 2017)

2. R Core Team. R: A language and environment for statistical computing. *R Foundation for Statistical Computing, Vienna, Austria* (2017). Available at: https://www.r-project.org. (Accessed: 1st February 2017)

3. Cartwright, J. Analysis of drought sensitivity in the Pacific Northwest (Washington, Oregon, and Idaho) from 2000 through 2016: U.S. Geological Survey data release. (2019). Available at: https://doi.org/10.5066/P9UNYG2R. (Accessed: 6th November 2019)

4. Franklin, S. *et al.* Building the United States National Vegetation Classification. *Ann. di Bot.* **2**, 1–9 (2012).

5. U.S. Geological Survey. GAP/LANDFIRE National Terrestrial Ecosystems dataset. *GAP/LANDFIRE National Terrestrial Ecosystems dataset* (2010). Available at: http://gapanalysis.usgs.gov/gaplandcover/featured-post-1/. (Accessed: 16th March 2017)

6. Eidenshink, J. *et al.* A project for monitoring trends in burn severity. *Fire Ecol.* **3**, 3–21 (2007).

7. U.S. Forest Service. Aerial insect and disease survey GIS data for Oregon and Washington 1947-present. *Forest Health Protection Program* (2016). Available at: https://www.fs.usda.gov/detail/r6/forest-grasslandhealth/insects-diseases/?cid=stelprd3791643. (Accessed: 1st December 2016)

8. Sims, D. A., Brzostek, E. R. & Rahman, A. F. An improved approach for remotely sensing water stress impacts on forest C uptake. *Glob. Chang. Biol.* 1–11 (2014). doi:10.1111/gcb.12537

9. Assal, T. J., Anderson, P. J. & Sibold, J. Spatial and temporal trends of drought effects in a heterogeneous semi-arid forest ecosystem. *For. Ecol. Manage.* **365**, 137–151 (2016).

10. Vogelmann, J. E., Tolk, B. & Zhu, Z. Monitoring forest changes in the southwestern United States using multitemporal Landsat data. *Remote Sens. Environ.* **113**, 1739–1748 (2009).

11. Clark, J. S. *et al.* The impacts of increasing drought on forest dynamics, structure, and biodiversity in the United States. *Glob. Chang. Biol.* **22**, 2329–2352 (2016).

12. Omernik, J. & Griffith, G. Ecoregions of the conterminous United States: evolution of a hierarchical spatial framework. *Environ. Manage.* **54**, 1249–1266 (2014).

13. Esri. ArcGIS Desktop. (2020). Available at: https://www.esri.com/en-us/arcgis/products/arcgis-desktop/overview. (Accessed: 8th January 2017)

14. Vicente-Serrano, S. M., Beguería, S. & López-Moreno, J. I. A multiscalar drought index sensitive to global warming: The standardized precipitation evapotranspiration index. *J. Clim.* **23**, 1696–1718 (2010).

15. Barnes, M. L. *et al.* Vegetation productivity responds to sub-annual climate conditions across semiarid biomes. *Ecosphere* **7**, e01339 (2016).

16. Vicente-Serrano, S. M. *et al.* Response of vegetation to drought time-scales across global land biomes. *Proc. Natl. Acad. Sci.* **110**, 52–57 (2013).

17. Abatzoglou, J. T., McEvoy, D. J. & Redmond, K. T. The west wide drought tracker: drought monitoring at fine spatial scales. *Bull. Am. Meteorol. Soc.* **98**, 1815–1820 (2017).

18. Yu, M., Li, Q., Hayes, M. J., Svoboda, M. D. & Heim, R. R. Are droughts becoming more frequent or severe in China based on the standardized precipitation evapotranspiration index: 1951-2010? *Int. J. Climatol.* **34**, 545–558 (2014).

19. Cavin, L. & Jump, A. Highest drought sensitivity and lowest resistance to growth suppression are found in the range core of the tree Fagus sylvatica L . not the equatorial range edge. *Glob. Chang. Biol.* **23**, 362–379 (2017).

20. Ahmadalipour, A., Moradkhani, H. & Svoboda, M. Centennial drought outlook over the CONUS using NASA-NEX downscaled climate ensemble. *Int. J. Climatol.* **37**, 2477–2491 (2017).

21. Hijmans, R., Phillips, S., Leathwick, J. & Elith, J. dismo: species distribution modeling, R package version 1.1-4. (2016). Available at: https://cran.r-project.org/web/packages/dismo/dismo.pdf. (Accessed: 1st May 2017)

22. Elith, J., Leathwick, J. R. & Hastie, T. A working guide to boosted regression trees. *J. Anim. Ecol.* **77**, 802–813 (2008).

23. Friedman, J., Hastie, T. & Tibshirani, R. Additive logistic regression: a statistical view of boosting. *Ann. Stat.* **28**, 337–407 (2000).

24. Fan, Y., Li, H. & Miguez-Macho, G. Global patterns of groundwater table depth. *Science (80-. ).* **339**, 940–943 (2013).

25. Tague, C. & Grant, G. E. Groundwater dynamics mediate low-flow response to global warming in snow-dominated alpine regions. *Water Resour. Res.* **45**, 1–12 (2009).

26. Kath, J. *et al.* Groundwater salinization intensifies drought impacts in forests and reduces refuge capacity. *J. Appl. Ecol.* **52**, 1116–1125 (2015).

27. McLaughlin, B. *et al.* Hydrologic refugia, plants and climate change. *Glob. Chang. Biol.* **23**, 1–21 (2017).
